# Tailored Nanogel Network Topology Enables Clinical Ultrasound‐Induced Mechanochemical Activation for In Vivo Therapy

**DOI:** 10.1002/anie.4591365

**Published:** 2026-05-25

**Authors:** Helin Li, Fangyin Song, Xiaoye Hu, Li Jing, Qi Shuai, Weike Su, Andrij Pich, Xin Li

**Affiliations:** ^1^ Collaborative Innovation Center of Yangtze River Delta Region Green Pharmaceuticals Zhejiang University of Technology Hangzhou China; ^2^ State Key Laboratory of Transvascular Implantation Devices, The Second Affiliated Hospital School of Medicine Zhejiang University Hangzhou China; ^3^ DWI‐Leibniz‐Institute for Interactive Materials Aachen Germany; ^4^ Institute for Technical and Macromolecular Chemistry RWTH Aachen University Aachen Germany; ^5^ Department of Medical Oncology, The Second Affiliated Hospital, School of Medicine Zhejiang University Hangzhou China; ^6^ Hepatobiliary and Pancreatic Interventional Treatment Center Division of Hepatobiliary and Pancreatic Surgery School of Medicine Zhejiang University Hangzhou China; ^7^ Aachen Maastricht Institute for Biobased Materials Maastricht University Geleen The Netherlands

**Keywords:** high‐frequency ultrasound, nanogels, network topology, polymer mechanochemistry, tumor therapy

## Abstract

Polymer mechanochemistry enables the selective mechanophore activation for precise molecular release. However, this field has long relied on the strong cavitation effects of low‐frequency ultrasound, which are incompatible with clinically high‐frequency ultrasound (> 1 MHz), hindering its biomedical applications. Herein, we report a nanogel network topology‐programmed mechanochemical strategy, enabling iterative activation of mechanophores under high‐frequency (up to 2.4 MHz) and low‐intensity (down to 2 W/cm^2^) ultrasound. By introducing network topology as a design parameter, we established the relationship between nanogel topology, ultrasound frequency, and mechanochemical activity, revealing that distinct network topologies selectively accumulate mechanical forces under different ultrasound frequencies. Meanwhile, the thermal effect of high‐frequency ultrasound lowers the activation barrier of mechanophores, further promoting their efficient activation. Remarkably, under high‐frequency ultrasound, nanogels achieve an activation rate of 12.6%/min, placing it among the most efficient activation systems reported to date. Importantly, combining nanogels with clinical low‐intensity focused ultrasound, we developed a new ultrasound‐mediated therapeutic paradigm—synergistic sonothermal and mechanochemical therapy, achieving complete elimination of advanced tumors with a single short treatment. This work establishes network topology engineering as a general strategy for programming mechanochemistry, and opens new opportunities for the in vivo biomedical applications of polymer mechanochemistry.

## Graphical abstract

The strategy of engineering nanogel network topology is developed to achieve iterative activation of mechanophores upon high‐frequency and low‐intensity ultrasound. This places the nanogel system among the systems that can respond to the ultrasound with the highest frequency and lowest intensity as well as have the fastest activation rate so far. The combination of nanogels with clinical ultrasound achieves a complete tumor elimination through synergistic sonothermal and mechanochemical therapy.

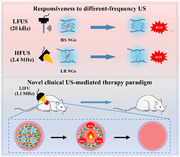

## 1 Introduction

Polymer mechanochemistry utilizes mechanical forces to selectively rearrange or cleave the mechanophores integrated into polymer systems [1]. Polymer mechanochemistry approaches has been widely applied in damage detection [2], optical sensing [3], self‐regulating materials [4], and controllable molecular activation and release [5]. Ultrasound (US) has been extensively used in clinical medicine, including US imaging [6], US‐targeted microbubble destruction [7], and high‐intensity focused US (HIFU) [8]. Moreover, US is the sole tool capable of transmitting mechanical force to the solutions. The combination of polymer mechanochemistry and US has paved the way for a new field of precision molecular release [5, 9, 10], addressing the challenge of non‐specific release of molecules in traditional US‐responsive systems [11]. However, this field is still in an early stage and its progress is slow, as two major challenges hinder its in vivo application [12, 13]. First, US‐mediated mechanophore cleavage and subsequent molecular release from these polymer systems are limited. This is because the dissociation of polymer systems caused by the mechanophore cleavage leads to the failure of the iteratively activated mechanophores. Second, the cleavage of most mechanophores depends on the strong cavitation effect of low‐frequency US (decades of kHz) and long US time (several hours). Nevertheless, these US parameters are not biosafe and are inconsistent with the inherent high‐frequency nature of clinical US equipment (> 1 MHz).

To increase the activation efficiency of mechanophores, knotted polymer systems and interlocked rotaxane architectures are designed to avoid the attachment of mechanophores to polymer backbone [14, 15, 16]. These ingenious designs enable iterative activation of mechanophores, leading to significant molecule release. In addition, optimizing the polymerization degree, molecular weight distribution, and shape (e.g., star‐shaped or three‐arm shaped) of polymer chains has been shown to enhance the activation efficiency of mechanophores [15, 17, 18, 19, 20, 21, 22, 23, 24, 25]. Moreover, the supramolecular assembly of polymer chains can enhance the mechanochemical activity of mechanophores [5, 16, 26, 27]. Compared with the dissolved state, the assembled state of polymers is more conducive to the accumulation and transmission of US force [28, 29, 30]. Nonetheless, existing studies still focus on linear polymer chains and their responsiveness under low‐frequency US (20 kHz). Meanwhile, the mechanophores still require prolonged US treatment (dozens of minutes) to be activated [31]. On the other hand, combining polymer systems with microbubbles can achieve mechanophore activation at higher US frequencies (hundreds of kHz) by the bubble rupture‐enhanced cavitation effect [32, 33]. Nevertheless, these US frequencies do not align with clinical US requirements, and the activation efficiency of these systems remains low (approximately 10%). Besides, the interaction between crosslinked network topology and mechanophore activation in polymer systems at various US frequencies is unexplored. To the best of our knowledge, the biomedical application of polymer mechanochemistry has not yet been validated in vivo, although the relevant studies have consistently emphasized its importance and necessity [12, 16].

**SCHEME 1:**
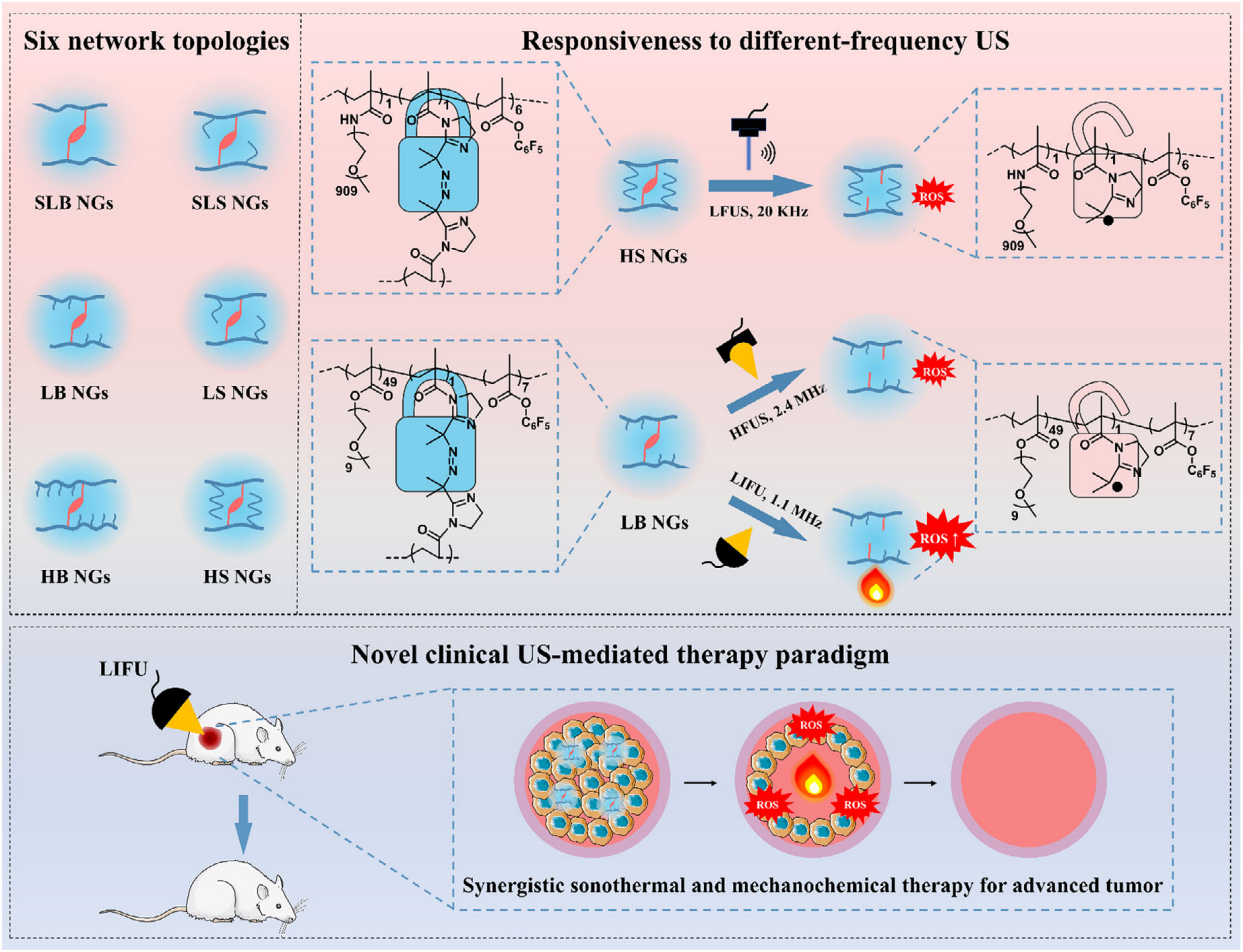
Schematic illustration of NG topology optimization, US responsiveness, and novel clinical US‐mediated therapy. Six amphiphilic polymers (SLB polymers: ∼12 kDa, LB polymers: ∼24 kDa, HB polymers: ∼42 kDa, SLS polymers: ∼12 kDa, LS polymers: ∼22 kDa, HS polymers: ∼42 kDa) are crosslinked with azo‐based mechanophores to form the NGs with different network topologies. These various NG topologies exhibit different responses to LFUS (20 kHz) and HFUS (2.4 MHz). HS NGs demonstrate the highest sensitivity to LFUS, while LB NGs show the highest sensitivity to HFUS. The thermal effect of LIFU increases the mechanochemical activity of mechanophores to promote their activation and reactive oxygen species (ROS) release. Under LIFU‐mediated synergistic therapy, the solid tumors with large volumes can be completely eliminated with just a single 2 min US treatment.

Herein, we developed nanogel (NG) network topology‐programmed mechanochemical strategy to realize the mechanophore response to high‐frequency US and subsequent iterative activation (Scheme 1). The optimized network topology facilitates the transduction of US force to the mechanophores, and the thermal effect of US increases their mechanochemical activity, achieving the activation upon high‐frequency and low‐intensity US. Besides, the crosslinked network prevents full dissociation post initial mechanophore activation, allowing for iterative activation. After US treatment for 5 min, the activation efficiency of mechanophores in NGs reaches about 63% (i.e., activation rate of 12.6%/min). This places the NG system among the systems that can respond to the US with the highest frequency and lowest intensity as well as have the highest activation rate so far (Table 1). Remarkably, we observed distinct response sensitivities of NGs with varying network topologies to low‐frequency US (LFUS, 20 kHz) and high‐frequency US (HFUS, 2.4 MHz). Single branched polymer‐crosslinked NGs exhibit the highest sensitivity to LFUS, while brush polymer‐crosslinked NGs possess the highest sensitivity to HFUS. Finally, by integrating the NG system with clinical low‐intensity focused US (LIFU), a novel US‐mediated therapeutic strategy, synergistic sonothermal and mechanochemical therapy, is developed. Notably, complete elimination of advanced tumors is achieved with only a single 2 min US treatment. Furthermore, the innovative LIFU‐mediated therapy can induce apoptosis and pyroptosis of tumor cells.

**TABLE 1:** Summary of US‐induced mechanochemical activation.

		US parameters						
Mechanophores	Carriers	Frequency	Intensity	Time	Activation efficiency	Activation rate	In vivo experiment	Ref.
Disulfide	Linear polymers	20 kHz	15.84 W**^.^** cm^−2^	240 min	80%	0.3%/min	No	[5]
Oxanorbornene	Linear polymers	20 kHz	8.2 W**^.^** cm^−2^	120 min	41%	0.3%/min	No	[54]
Norborn‐2‐en‐7‐one	Linear polymers with multi‐mechanophores	20 kHz	8.8 W**^.^** cm^−2^	240 min	58%	0.2%/min	No	[55]
Rotaxane	Linear polymers with multi‐mechanophores	20 kHz	13 W**^.^** cm^−2^	90 min	69%	0.8%/min	No	[16]
Azo	Hydrogels	550 kHz	115 W**^.^** cm^−2^	72 h	10%	0.1%/h	No	[9]
Disulfide	Microbubble with polymer shell	680 kHz	10 W**^.^** cm^−2^	5 min	10%	1.9%/min	No	[33]
Oxanorbornene	Linear polymers + gas vesicles	330 kHz	3.6 W**^.^** cm^−2^	10 min	8%	0.8%/min	No	[32]
**Azo**	**Nanogels with different topologies**	**2.4 MHz**	**2 W^.^cm^−2^**	**5 min**	**63%**	**12.6%/min**	**Yes**	**This work**

## 2 Results and Discussion

### 2.1 Synthesis and Characterization of NGs With Different Network Topologies

We first synthesized six amphiphilic copolymers with various topological structures through RAFT polymerization or covalent linkage, namely super‐low molecular weight brush polymers (SLB polymers, ∼12 kDa, Figures S1–S8), low molecular weight brush polymers (LB polymers, ∼24 kDa, Figures S9–S14), high molecular weight brush polymers (HB polymers, ∼42 kDa, Figures S15–S20), super‐low molecular weight single branched polymers (SLS polymers, ∼12 kDa, Figures S21–S26), low molecular weight single branched polymers (LS polymers, ∼22 kDa, Figures S27–S32), and high molecular weight single branched polymers (HS polymers, ∼42 kDa, Figures S33–S38). The hydrophilic chain was biocompatible polyethylene glycol (PEG) or its analogue poly(ethylene glycol) methyl ether methacrylate (PEGMEMA), and the hydrophobic chain was poly(pentafluorophenyl methacrylate) (PPFPMA) which can be used for further crosslinking and modification. To achieve higher mechanochemical activity, azo‐based agents (AIPH) with low bond energy were chosen as crosslinkers among numerous mechanophores [34, 35]. Through the self‐assembly and crosslinking of reactive copolymers (Figures S39 and 40) [36, 37], the corresponding NGs with six network topologies were synthesized and named accordingly to the corresponding building blocks SLB NGs, LB NGs, HB NGs, SLS NGs, LS NGs, and HS NGs. The DLS and TEM images showed that the resulting NGs exhibit the regular spherical shape and the average hydrodynamic diameter of 100–300 nm (Figure 1). Moreover, the corresponding non‐crosslinked polymer system bearing AIPH (i.e., LB‐A polymers) was synthesized as a control group (Figures S41–S42). Besides, a traditional US‐activatable linear polymer (PAP, ∼40 kDa) was prepared as a control group (Figures S43–S45).

**FIGURE 1:**
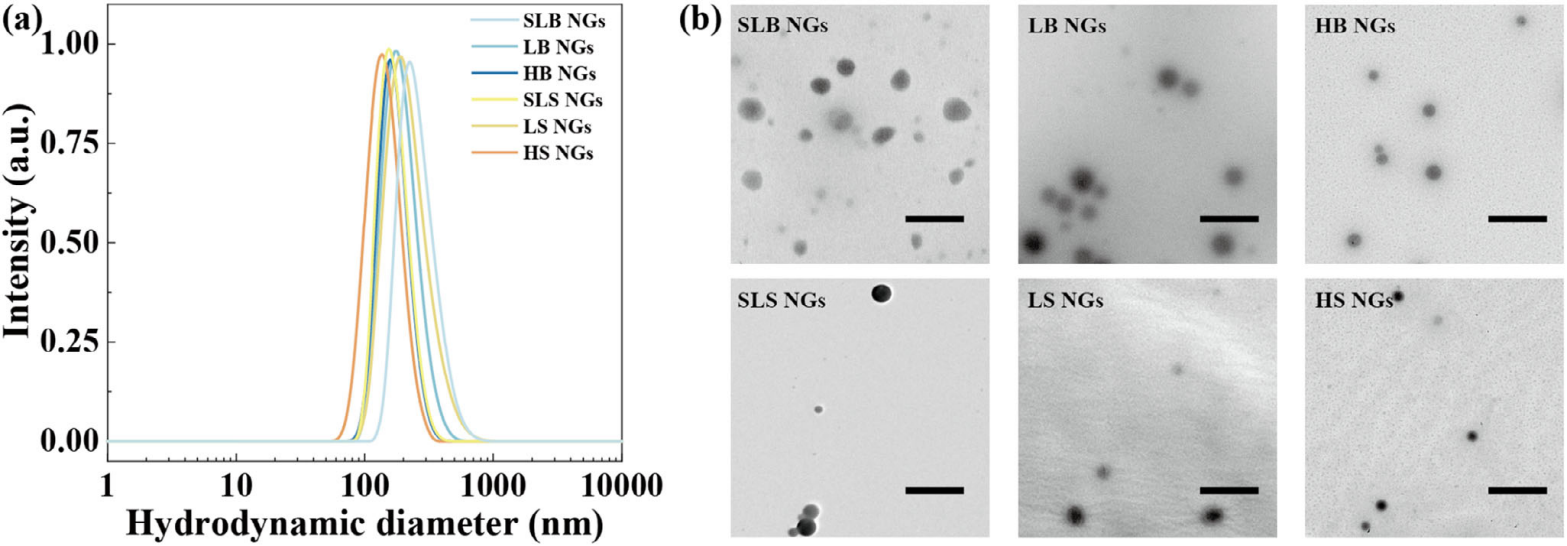
Morphology and size of NGs. (a) Hydrodynamic diameter distribution and (b) TEM images of SLB NGs, LB NGs, HB NGs, SLS NGs, LS NGs, and HS NGs. Scale bar 500 nm.

### 2.2 Mechanophore Activation Upon LFUS

**FIGURE 2:**
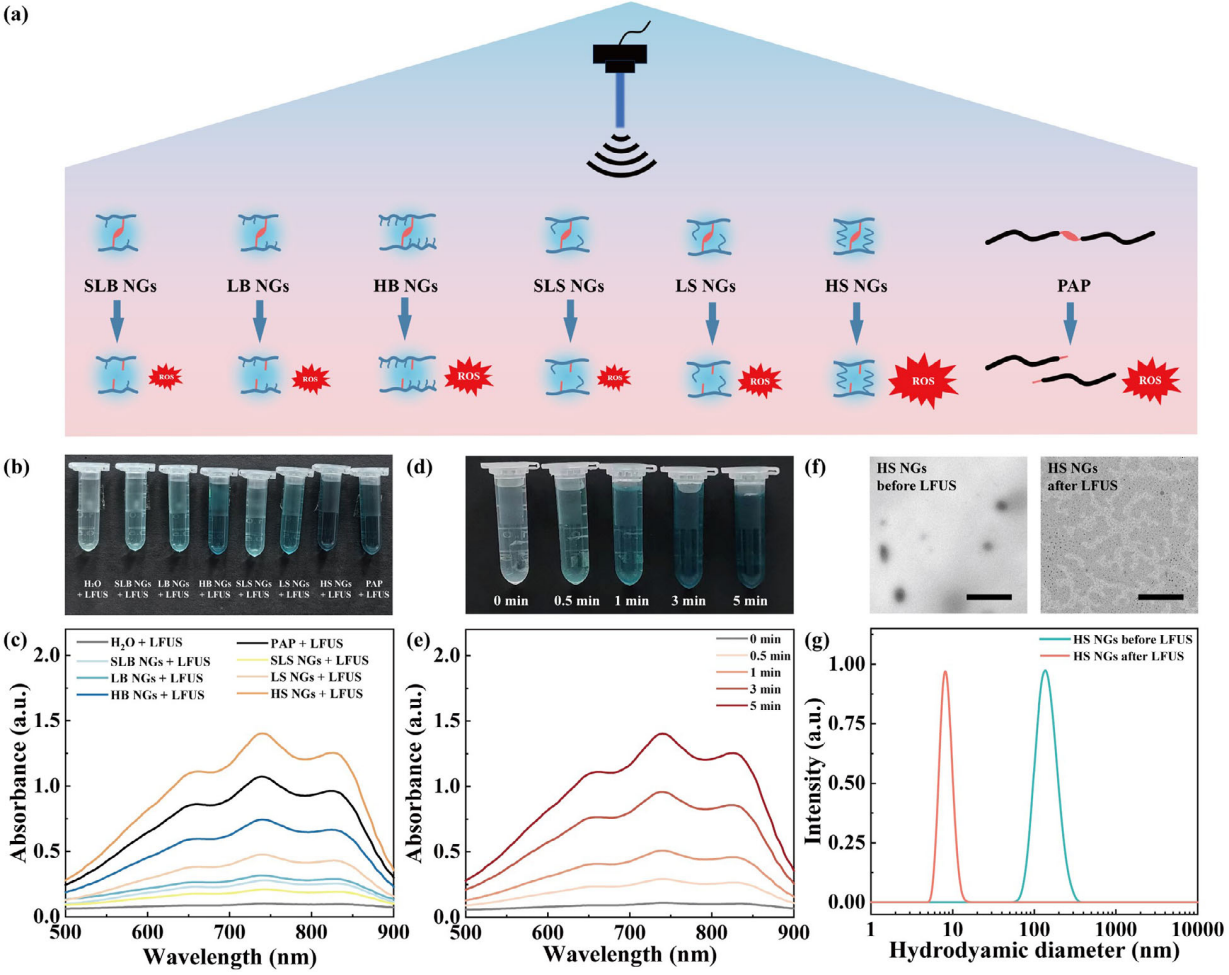
Mechanophore activation of NGs upon LFUS. (a) LFUS responsiveness and radical generation of NGs and PAP. (b) Photographs and (c) UV–vis spectra of H_2_O, PAP, and NG solutions containing ABTS upon LFUS for 5 min (20 kHz). (d) Photographs and (e) UV–vis spectra of HS NG solutions containing ABTS upon LFUS at different times (0–5 min). (f) TEM images and (g) hydrodynamic diameter distribution of HS NGs before and after LFUS. Scale bar 500 nm.

The mechanochemical activities of azo‐based mechanophores in these NGs were investigated. Upon LFUS (20 kHz), the molecular weight of most PAP was reduced to half of the original value (Figure S46), which is consistent with previous literature [38]. The strong cavitation effect of LFUS can activate the azo cleavage in traditional linear polymers. After US‐mediated cleavage, the azo‐based mechanophores form alkyl radicals, which are further converted into ROS in aqueous environment (Figure S47) [9, 39]. In the 2,2'‐azinobis(3‐ethylbenzothiazoline‐6‐sulfonic acid) diammonium salt (ABTS) assay (Figures S48‐49 **),** we found that not all types of network topologies of NGs can promote the activation of mechanophores (Figure 2a–c) [40, 41]. Compared to PAP, only HS NGs showed a significantly increased activation of mechanophores. After treatment for 5 min, approximately 59% of mechanophores in HS NGs were activated (Figures 2d,e, S50, and S51). During US treatment, the temperature of NG solution increased by only 1.3°C, suggesting that LFUS primarily exhibits a mechanical effect with negligible thermal effect (Figure S52). Moreover, the activation of these mechanophores resulted in the degradation of NGs (Figure 2f,g), which may facilitate their metabolism in vivo [42].

### 2.3 Mechanophore Activation Upon HFUS

**FIGURE 3:**
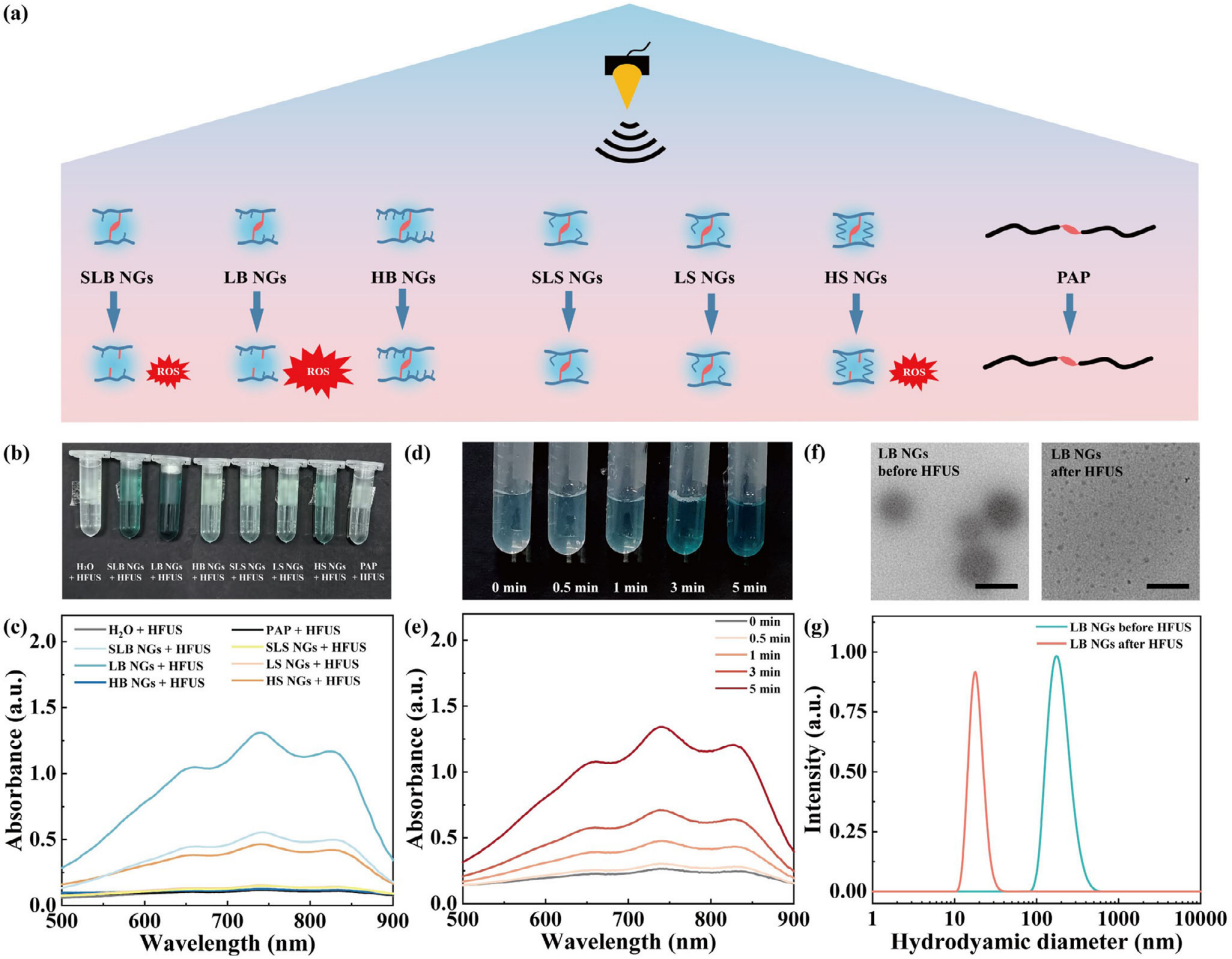
Mechanophore activation of NGs upon HFUS. (a) HFUS responsiveness and ROS generation of NGs and PAP. (b) Photographs and (c) UV–vis spectra of H_2_O, PAP, and NG solutions containing ABTS upon HFUS for 5 min (2.4 MHz). (d) Photographs and (e) UV–vis spectra of LB NG solutions containing ABTS upon HFUS at different times (0–5 min). (f) TEM images and (g) hydrodynamic diameter distribution of LB NGs before and after HFUS treatment. Scale bar 200 nm.

Furthermore, biocompatible HFUS (2.4 MHz) was adopted to treat the azo‐based mechanophores of NGs. Notably, the weak cavitation effect of HFUS was unable to activate the mechanophores in PAP (Figure 3a–c). Excitingly, the mechanophores in LB NGs, SLB NGs, and HS NGs could be effectively activated by HFUS. These findings indicated that the optimized topological structure and crosslinked network are conducive to the force accumulation of HFUS on the mechanophores, a phenomenon unattainable in traditional linear polymers. After treatment for 5 min, LB NGs possessed the highest activation efficiency at approximately 63%, corresponding to a remarkable activation rate of about 12.6%/min (Figures 3d,e, S53, and 54), after which they underwent degradation (Figure 3f,g). To exclude the influence of thermal effect of HFUS on mechanophore activation, the temperature change of NG solution upon HFUS was monitored (Figure S55). Clearly, the maximum temperature of NG solution at HFUS focus was about 54.3°C which is insufficient to directly activate the mechanophores (Figure S56). In addition, upon HFUS, LB‐A polymers exhibited only negligible radical generation (Figure S57), demonstrating that the mechanophore activation in NGs is driven by the mechanical effect of US accumulation through the network topology optimization, rather than via thermal effect. These results revealed that through a facile engineering of NG network topology, the mechanophores can be endowed with efficient responsiveness and iterative activation to high‐frequency US (> 1 MHz). Notably, even at a low US intensity of 2 W/cm^2^, HFUS could also rapidly activate the mechanophores in NGs (Figures S58 and S59). After treatment for 3 min, the activation efficiency of mechanophores in LB NGs was about 14% (Figures S60 and S61). Therefore, the developed NGs represent a class of systems capable of responding to the highest US frequency (2.4 MHz) and lowest US intensity (2 W/cm^2^), while exhibiting the highest activation rate (12.6%/min).

### 2.4 Comparison of Mechanophore Activation Upon Low‐Frequency and High‐Frequency US

By comparing the mechanophore activation in NGs, we observed the distinct sensitivities of these NGs with different network topologies to LFUS (20 kHz) and HFUS (2.4 MHz). Although the influence of polymer topology on the mechanophore activity has been studied using low‐frequency US, it is challenging to induce mechanophore activation using high‐frequency US (> 1 MHz). When using the clinical US equipment, further investigation is needed to explore the relationship between network topology and mechanophore activation. Comparing the experimental data (Figures 2 and 3), we found that the activation efficiency of mechanophores in the NGs is jointly determined by both the polymerization degree and the topological structure of polymers. Upon LFUS (Figure 2b,c), the activation efficiency of both brush polymer‐crosslinked and single branched polymer‐crosslinked NGs increased with the polymerization degree (Figure S50), aligning with the conclusion of previous literature [31, 43]. Moreover, compared to brush polymer‐crosslinked NGs (SLB NGs: 8%, LB NGs: 10%, HB NGs: 28%), single branched polymer‐crosslinked NGs with same polymerization degree exhibited a higher activation efficiency (SLS NGs: 9%, LS NGs: 16%, HS NGs: 59%) (Figure S50). In contrast, upon HFUS (Figure 3b,c), the activation efficiency of single branched polymer‐crosslinked NGs also increased with the polymerization degree (SLS NGs: 2%, LS NGs: 3%, HS NGs: 11%) (Figure S53). However, the HB NGs in brush polymer‐crosslinked NGs exhibited a significantly reduced activation efficiency (SLB NGs: 11%, LB NGs: 63%, HB NGs: 2%) (Figure S53). This is mainly because the increase of brush polymer chains in HB NGs disperses the force along the mechanophore backbone, thereby preventing effective activation. Additionally, except for HB NGs, brush polymer‐crosslinked NGs showed a higher activation efficiency compared to single branched polymer‐crosslinked NGs. These results indicated that the force accumulation effect within the NGs differed substantially under different US frequencies: single branched polymer‐crosslinked NGs exhibited higher sensitivity to LFUS (HS NGs: 59%), whereas brush polymer‐crosslinked NGs are more sensitive to HFUS (LB NGs: 63%).

### 2.5 HFUS‐Mediated Mechanochemical Therapy of Tumors In Vivo

**FIGURE 4:**
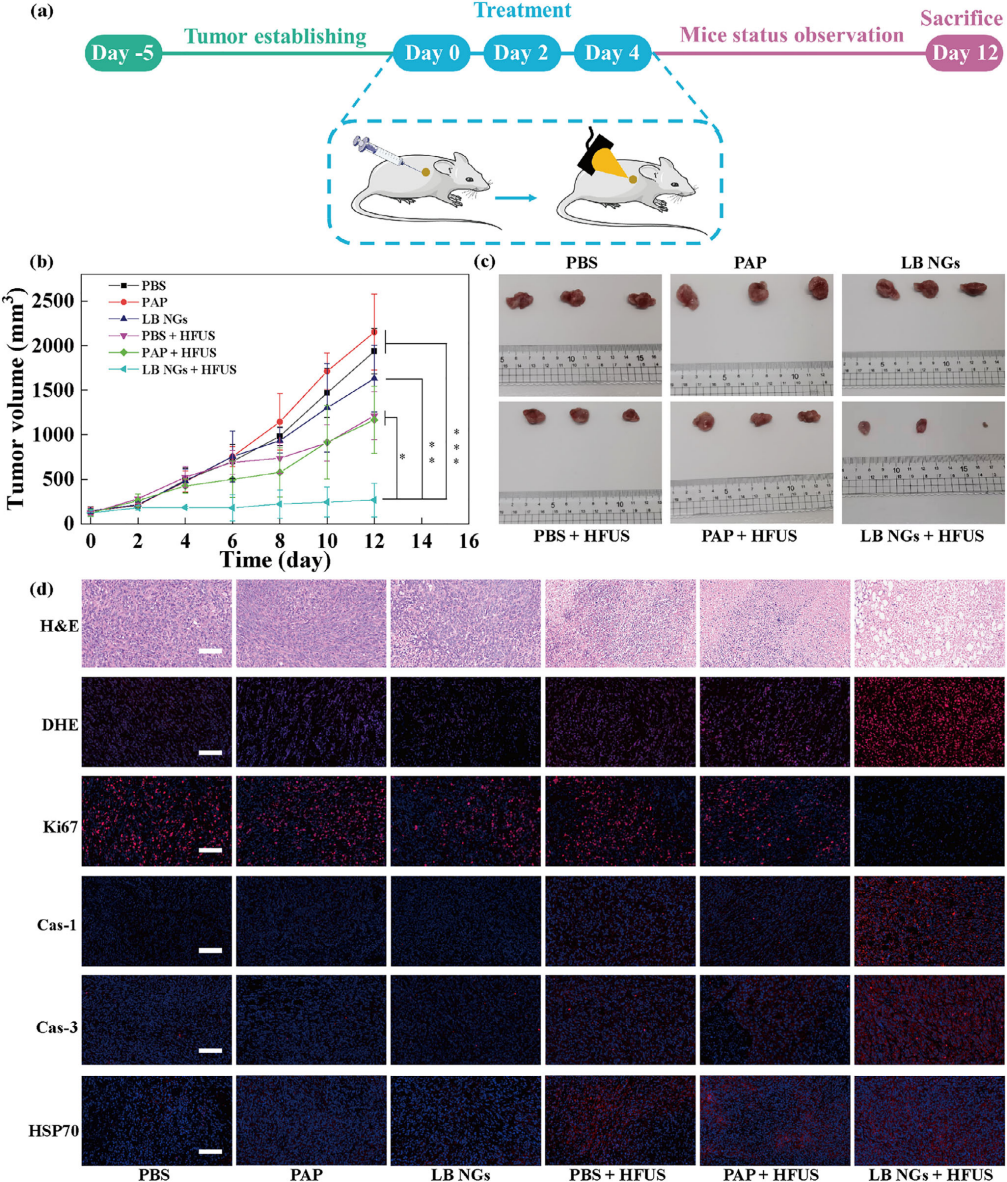
HFUS‐mediated tumor therapy in vivo. (a) Schematic illustration of administration and HFUS process used for tumor treatment. (b) Relative tumor variation of mice as a function of time in different groups (*n *= 3). (c) Photographs of the extracted tumors on the last day after different treatments. (d) DHE, H&E, Ki67, Cas‐1, Cas‐3, and HSP70 staining of tumor slices in different groups (scale bar: 100 µm).

Our strategy achieves highly efficient iterative activation of mechanophores upon a biocompatible high‐frequency US and solves the challenges of in vivo application of polymer mechanochemistry. Next, we explored HFUS‐mediated tumor therapy in vivo. First, the thermal effect of HFUS on mice was monitored (Figure S62). After HFUS treatment, the temperature of tumor in mice reached 41.6°C, at which only limited ablation efficacy was achieved due to the protective effect of heat shock proteins (HSP70) (Figure 4d) [44, 45]. Therefore, HFUS‐mediated mechanophore activation and ROS production, that is, mechanochemical therapy, play pivotal roles in tumor therapy. Additionally, nude mice bearing 4T1 tumor were randomly divided into six groups with different treatments: 1) PBS, 2) PAP, 3) LB NGs, 4) PBS + HFUS, 5) PAP + HFUS, and 6) LB NGs + HFUS. The treatment timeline was given in Figure 4a. Compared to PBS group, the tumor growth trends in PAP and LB NGs groups were similar, suggesting that these samples exhibit good biocompatibility (Figure 4b). A moderate level of anti‐tumor effect was observed in PBS + HFUS and PAP + HFUS groups, while the significant tumor suppression was achieved in LB NGs + HFUS group (Figures 4b,c and S63). Besides, the DHE and H&E staining in tumor slices demonstrated that compared with other groups, a significant production of ROS and a large amount of tumor cell necrosis were observed in LB NGs + HFUS group (Figure 4d). The Ki67 staining showed that the proliferation of tumor cells was obviously inhibited. These results revealed that HFUS‐mediated mechanochemical therapy exhibits an excellent anti‐tumor efficacy in vivo. Moreover, the Cas‐1 and Cas‐3 staining suggested that the ROS production induced by mechanochemical therapy could lead to tumor cell death through pyroptosis and apoptosis. It is worth mentioning that our work is the first to match biocompatible high‐frequency US with polymer mechanochemistry for tumor therapy in vivo. Excitingly, compared to traditional sonodynamic therapy [46], mechanochemical therapy does not rely on sonosensitizers and is not limited by the hypoxic tumor microenvironment.

### 2.6 Clinical LIFU‐Mediated Mechanophore Activation and Synergistic Therapy of Tumors

**FIGURE 5:**
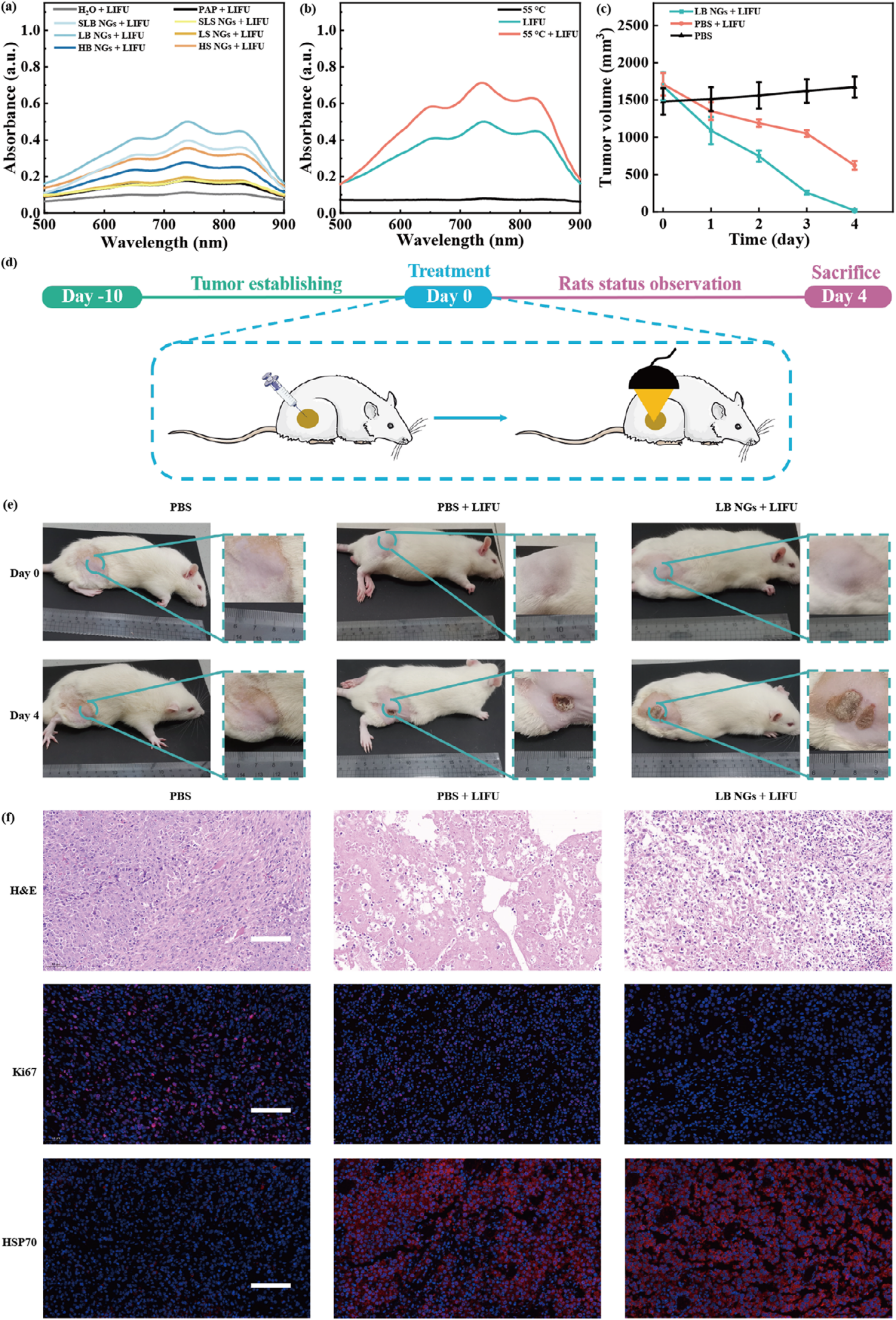
Mechanophore activation of NGs and synergistic therapy of tumors upon LIFU (1.1 MHz). (a) UV–vis spectra of H_2_O, PAP, and NG solutions containing ABTS upon LIFU for 5 min. (b) UV–vis spectra of LB NGs solution containing ABTS at different treatment groups. (c) Relative tumor variation of rats over time in various groups (*n *= 3). (d) Schematic illustration of administration and LIFU process used for the treatment of advanced tumors in rats. (e) Photographs of the rats bearing tumor on the last day after different treatments. (f) H&E, Ki67, and HSP70 staining of tumor slices in different groups (scale bar: 100 µm).

High‐intensity focused ultrasound (HIFU, > 100 W/cm^2^) is widely applied in clinical practice [47]. Compared to other hyperthermia technologies, HIFU‐mediated sonothermal therapy possesses some unique advantages, including no bleeding during treatment, low infection rate, fewer complications, and enhanced immune function of the body [10, 48]. This is because small focal spot and high intensity of HIFU enable the tumor temperature to rapidly rise above 65°C, almost unaffected by blood flow, thus achieving the effect of rapid coagulation necrosis of tumors [49, 50, 51]. However, excessive US intensity and thermal effect can also cause irreversible damage to human skin and normal tissue surrounding tumors [52, 53]. Therefore, we chose clinical LIFU (1.1 MHz, 64 W/cm^2^) to evaluate the mechanochemical activity of mechanophores in NGs and their potential for tumor therapy in vivo (Figures S64 and S65). Apparently, the mechanophores in LB NGs exhibited the highest sensitivity to LIFU (Figure 5a). After treatment for 5 min, the activation efficiency of mechanophores reached about 19% (Figure S66).

Next, the influence of thermal effect of LIFU on mechanophore activation was investigated. After the treatment, the temperature of tumor in rats exceeds 50°C (Figures S67 and S68), indicating that LIFU‐mediated thermal effect can be used for tumor ablation. Although the aforementioned results have demonstrated that the thermal effect alone is insufficient to cleave the mechanophores in NGs directly, it can improve the mechanochemical activity of mechanophores, thereby significantly increasing mechanical effect‐mediated activation efficiency (about 31%) (Figures 5b, S69, and S70). Therefore, the combined thermal and mechanical effects of LIFU synergistically enhance the activation efficiency of mechanophores.

Finally, we constructed a large tumor in rat to simulate advanced tumor for the validation of therapeutic efficacy of LIFU‐mediated synergistic sonothermal and mechanochemical therapy. Encouragingly, in LB NGs + LIFU group, a complete elimination of tumor was achieved with just one treatment of LIFU for 2 min (Figure 5c,d). In PBS + LIFU group, tumor destruction was observed to some extent due to the thermal effect of LIFU, although complete remission was not achieved. Moreover, H&E and Ki67 staining revealed significant tumor cell necrosis and tumor proliferation inhibition in LB NGs + LIFU group, respectively (Figure 5e). These results demonstrated that compared to LIFU therapy alone, LIFU‐mediated synergistic therapy exerts enhanced efficacy in tumor treatment and are expected to achieve a complete elimination of advanced tumors.

## 3 Conclusion

Overall, we have demonstrated that high‐frequency US response and iterative activation of mechanophores can be achieved simultaneously by engineering the NG network topology. Upon high‐frequency US, the optimized network topology was conducive to force accumulation of US on the mechanophores, meanwhile the thermal effect of US can increase their mechanochemical activity, thereby achieving the activation under weak cavitation effect (frequency up to 2.4 MHz, intensity down to 2 W/cm^2^). Moreover, the crosslinked network prevented the complete dissociation of systems after initial mechanophore activation, thus enabling further iterative activation. After high‐frequency US treatment, the activation rate of mechanophores in NGs reached about 12.6%/min, placing it among the most efficient activation systems reported to date. Additionally, the relationship between nanogel topology, ultrasound frequency, and mechanochemical activity was established. HS NGs exhibit the highest sensitivity to LFUS, while LB NGs possess the highest sensitivity to HFUS. More importantly, the combination of the NG system with clinical LIFU achieved the complete elimination of advanced tumors with just a single US treatment for 2 min by synergistic sonothermal and mechanochemical therapy. This work not only paves the way for the in vivo application of polymer mechanochemistry but also provides a novel paradigm for clinical US‐mediated precision tumor therapy.

## Conflicts of Interest

The authors declare no conflicts of interest.

## Supporting information




**Supporting File**: anie72788‐sup‐0001‐SuppMat.docx

## Data Availability

The data that support the findings of this study are available from the corresponding author upon reasonable request.

## References

[1] R. T. O'Neill and R. Boulatov , “The Many Flavours of Mechanochemistry and Its Plausible Conceptual Underpinnings,” Nature Reviews Chemistry 5 (2021): 148–167, 10.1038/s41570-020-00249-y. 37117533

[2] M. A. Ghanem , A. Basu , R. Behrou , et al., “The Role of Polymer Mechanochemistry in Responsive Materials and Additive Manufacturing,” Nature Reviews Materials 6 (2021): 84–98, 10.1038/s41578-020-00249-w.

[3] A. C. Overholts , W. G. Razo , and M. J. Robb , “Mechanically Gated Formation of Donor–acceptor Stenhouse Adducts Enabling Mechanochemical Multicolour Soft Lithography,” Nature Chemistry 15 (2023): 332–338, 10.1038/s41557-022-01126-5. 36690834

[4] T. Matsuda , R. Kawakami , R. Namba , T. Nakajima , and J. P. Gong , “Mechanoresponsive Self‐growing Hydrogels Inspired by Muscle Training,” Science 363 (2019): 504–508, 10.1126/science.aau9533. 30705187

[5] S. Huo , P. Zhao , Z. Shi , et al., “Mechanochemical Bond Scission for the Activation of Drugs,” Nature Chemistry 13 (2021): 131–139, 10.1038/s41557-020-00624-8. 33514936

[6] A. Farhadi , G. H. Ho , D. P. Sawyer , R. W. Bourdeau , and M. G. Shapiro , “Ultrasound Imaging of Gene Expression in Mammalian Cells,” Science 365 (2019): 1469–1475, 10.1126/science.aax4804. 31604277 PMC6860372

[7] H. Huang , Y. Zheng , M. Chang , et al., “Ultrasound‐Based Micro‐/Nanosystems for Biomedical Applications,” Chemical Reviews 124 (2024): 8307–8472, 10.1021/acs.chemrev.4c00009. 38924776

[8] Y. Meng , K. Hynynen , and N. Lipsman , “Applications of Focused Ultrasound in the Brain: From Thermoablation to Drug Delivery,” Nature Reviews Neurology 17 (2021): 7–22, 10.1038/s41582-020-00418-z. 33106619

[9] G. Kim , Q. Wu , J. L. Chu , et al., “Ultrasound Controlled Mechanophore Aactivation in Hydrogels for Cancer Therapy,” Proceedings of the National Academy of Sciences of the United States of America 119 (2022): e2109791119. 35046028 10.1073/pnas.2109791119 PMC8795563

[10] M. A. O'Reilly , “Exploiting the Mechanical Effects of Ultrasound for Noninvasive Therapy,” Science (New York, NY) 385 (2024): eadp7206–eadp7206, 10.1126/science.adp7206. 39265013

[11] Y. Zhang , J. Yu , H. N. Bomba , Y. Zhu , and Z. Gu , “Mechanical Force‐Triggered Drug Delivery,” Chemical Reviews 116 (2016): 12536–12563, 10.1021/acs.chemrev.6b00369. 27680291

[12] R. Boulatov , “The Liberating Force of Ultrasound,” Nature Chemistry 13 (2021): 112–114, 10.1038/s41557-020-00623-9. 33514931

[13] B. A. Versaw , T. Zeng , X. Hu , and M. J. Robb , “Harnessing the Power of Force: Development of Mechanophores for Molecular Release,” Journal of the American Chemical Society 143 (2021): 21461–21473, 10.1021/jacs.1c11868. 34927426

[14] T. Zeng , L. A. Ordner , P. Liu , and M. J. Robb , “Multimechanophore Polymers for Mechanically Triggered Small Molecule Release With Ultrahigh Payload Capacity,” Journal of the American Chemical Society 146 (2023): 95–100, 10.1021/jacs.3c11927. 38157405 PMC10786027

[15] E. M. Lloyd , J. R. Vakil , Y. Yao , N. R. Sottos , and S. L. Craig , “Covalent Mechanochemistry and Contemporary Polymer Network Chemistry: A Marriage in the Making,” Journal of the American Chemical Society 145 (2023): 751–768, 10.1021/jacs.2c09623. 36599076

[16] L. Chen , R. Nixon , and G. De Bo , “Force‐controlled Release of Small Molecules With a Rotaxane Actuator,” Nature 628 (2024): 320–325, 10.1038/s41586-024-07154-0. 38600268 PMC11006608

[17] M. E. McFadden and M. J. Robb , “Force‐Dependent Multicolor Mechanochromism From a Single Mechanophore,” Journal of the American Chemical Society 141 (2019): 11388–11392, 10.1021/jacs.9b05280. 31282668

[18] P. A. May , N. F. Munaretto , M. B. Hamoy , M. J. Robb , and J. S. Moore , “Is Molecular Weight or Degree of Polymerization a Better Descriptor of Ultrasound‐Induced Mechanochemical Transduction?,” ACS Macro Letters 5 (2016): 177–180, 10.1021/acsmacrolett.5b00855. 35614695

[19] D. C. Church , G. I. Peterson , and A. J. Boydston , “Comparison of Mechanochemical Chain Scission Rates for Linear Versus Three‐Arm Star Polymers in Strong Acoustic Fields,” ACS Macro Letters 3 (2014): 648–651, 10.1021/mz5003068. 35590762

[20] Y. Lin , Y. Zhang , Z. Wang , and S. L. Craig , “Dynamic Memory Effects in the Mechanochemistry of Cyclic Polymers,” Journal of the American Chemical Society 141 (2019): 10943–10947, 10.1021/jacs.9b03564. 31283207

[21] T. Watabe and H. Otsuka , “Enhancing the Reactivity of Mechanically Responsive Units via Macromolecular Design,” Macromolecules 57 (2024): 425–433, 10.1021/acs.macromol.3c01280.

[22] H. Yokochi , M. Ohira , M. Oka , et al., “Topology Transformation Toward Cyclic, Figure‐Eight‐Shaped, and Cross‐Linked Polymers Based on the Dynamic Behavior of a Bis(hindered amino)Disulfide Linker,” Macromolecules 54 (2021): 9992–10000, 10.1021/acs.macromol.1c01437.

[23] H. Zhang and C. E. Diesendruck , “Linear Versus Helical Side Chains in Bottlebrush Polymers: Morphology and Mechanochemistry,” Macromolecules 57 (2024): 3664–3670, 10.1021/acs.macromol.3c02573.

[24] H. Zhang and C. E. Diesendruck , “Accelerated Mechanochemistry in Helical Polymers,” Angewandte Chemie International Edition 61 (2022): e202115325, 10.1002/anie.202115325. 35075760 PMC9303913

[25] G. I. Peterson and T.‐L. Choi , “The Influence of Polymer Architecture in Polymer Mechanochemistry,” Chemical Communications 57 (2021): 6465–6474, 10.1039/D1CC02501E. 34132272

[26] R. Küng , A. Germann , M. Krüsmann , et al., “Mechanoresponsive Metal‐organic Cage‐crosslinked Polymer Hydrogels.” Chemistry—A European Journal 29 (2023): e202300079. 36715238 10.1002/chem.202300079

[27] R. Küng , T. Pausch , D. Rasch , R. Göstl , and B. M. Schmidt , “Mechanochemical Release of Non‐Covalently Bound Guests From a Polymer‐Decorated Supramolecular Cage.” Angewandte Chemie International Edition 60 (2021): 13626–13630.. 33729649 10.1002/anie.202102383 PMC8251918

[28] H. Li , R. Göstl , M. Delgove , et al., “Promoting Mechanochemistry of Covalent Bonds by Noncovalent Micellar Aggregation,” ACS Macro Letters 5 (2016): 995–998, 10.1021/acsmacrolett.6b00579. 35614648

[29] Q. Li , Y.‐X. Wang , and Y. Chen , “Unraveling Ultrasonic Stress Response of Nanovesicles by the Mechanochromism of Self‐Assembled Polydiacetylene,” ACS Macro Letters 11 (2022): 103–109, 10.1021/acsmacrolett.1c00715. 35574789

[30] H. Hu , Y. Zhou , B. Xi , and Y. Li , “Polymer Mechanochemistry in Confined Spaces,” Angewandte Chemie International Edition 64 (2025): e202417357, 10.1002/anie.202417357. 39365280

[31] L. Tu , Z. Liao , Z. Luo , Y.‐L. Wu , A. Herrmann , and S. Huo , “Ultrasound‐Controlled Drug Release and Drug Activation for Cancer Therapy,” Exploration 1 (2021): 20210023, 10.1002/EXP.20210023. 37323693 PMC10190934

[32] Y. Yao , M. E. McFadden , S. M. Luo , et al., “Remote Control of Mechanochemical Reactions Under Physiological Conditions Using Biocompatible Focused Ultrasound,” Proceedings of the National Academy of Sciences of the United States of America 120 (2023): e2309822120, 10.1073/pnas.2309822120. 37725651 PMC10523651

[33] M. Xuan , J. Fan , V. N. Khiem , et al., “Polymer Mechanochemistry in Microbubbles,” Advanced Materials 35 (2023): e2305130, 10.1002/adma.202305130. 37494284

[34] Y. Li , B. Xue , J. Yang , et al., “Azobenzene as A Photoswitchable Mechanophore,” Nature Chemistry 16 (2024): 16151–16157. 10.1038/s41557-023-01389-6 38052946

[35] J. Li , C. Nagamani , and J. S. Moore , “Polymer Mechanochemistry: From Destructive to Productive,” Accounts of Chemical Research 48 (2015): 2181–2190, 10.1021/acs.accounts.5b00184. 26176627

[36] M. Zou , P. Zhao , J. Fan , R. Goestl , and A. Herrmann , “Microgels as Drug Carriers for Sonopharmacology,” Journal of Polymer Science 60 (2022): 1864–1870, 10.1002/pol.20210874.

[37] P. J. Roth , T. P. Davis , and A. B. Lowe , “UCST‐driven Self‐assembly and Crosslinking of Diblock Copolymer Micelles,” Polymer Chemistry 3 (2012): 2228–2235, 10.1039/c2py20204b.

[38] K. L. Berkowski , S. L. Potisek , C. R. Hickenboth , and J. S. Moore , “Ultrasound‐Induced Site‐Specific Cleavage of Azo‐Functionalized Poly(ethylene glycol),” Macromolecules 38 (2005): 8975–8978, 10.1021/ma051394n.

[39] H. T. Baytekin , B. Baytekin , and B. A. Grzybowski , “Mechanoradicals Created in “Polymeric Sponges” Drive Reactions in Aqueous Media,” Angewandte Chemie‐International Edition 51 (2012): 3596–3600, 10.1002/anie.201108110. 22383092

[40] E. P. Labrinea and C. A. Georgiou , “Stopped‐flow Method for Assessment of pH and Timing Effect on the ABTS Total Antioxidant Capacity Assay,” Analytica Chimica Acta 526 (2004): 63–68, 10.1016/j.aca.2004.09.040.

[41] I. R. Ilyasov , V. L. Beloborodov , I. A. Selivanova , and R. P. Terekhov , “ABTS/PP Decolorization Assay of Antioxidant Capacity Reaction Pathways,” International Journal of Molecular Sciences 21 (2020): 1131, 10.3390/ijms21031131. 32046308 PMC7037303

[42] F. Xu , J. Zhu , L. Lin , et al., “Multifunctional PVCL Nanogels With Redox‐responsiveness Enable Enhanced MR Imaging and Ultrasound‐promoted Tumor Chemotherapy,” Theranostics 10 (2020): 4349–4358, 10.7150/thno.43402. 32292499 PMC7150492

[43] A. Levy , E. Gaver , F. Wang , O. Galant , and C. E. Diesendruck , “The Effect of Intramolecular Cross Links on the Mechanochemical Fragmentation of Polymers in Solution,” Chemical Communications 53 (2017): 10132–10135, 10.1039/C7CC04885H. 28848956

[44] K. Richter , M. Haslbeck , and J. Buchner , “The Heat Shock Response: Life on the Verge of Death,” Molecular Cell 40 (2010): 253–266, 10.1016/j.molcel.2010.10.006. 20965420

[45] E. M. Knavel and C. L. Brace , “Tumor Ablation: Common Modalities and General Practices,” Techniques in Vascular and Interventional Radiology 16 (2013): 192–200, 10.1053/j.tvir.2013.08.002. 24238374 PMC4281168

[46] S. Sun , X. Huang , N. Yang , et al., “Fluorinated Titanium Oxide (TiO2–xFx) Nanospindles as Ultrasound‐Triggered Pyroptosis Inducers to Boost Sonodynamic Immunotherapy,” ACS Nano 18 (2024): 19756–19770. 10.1021/acsnano.4c05448 39010657

[47] V. S. Bachu , J. Kedda , I. Suk , J. J. Green , and B. Tyler , “High‐Intensity Focused Ultrasound: A Review of Mechanisms and Clinical Applications,” Annals of Biomedical Engineering 49 (2021): 1975–1991, 10.1007/s10439-021-02833-9. 34374945 PMC8608284

[48] Z. Izadifar , Z. Izadifar , D. Chapman , and P. Babyn , “An Introduction to High Intensity Focused Ultrasound: Systematic Review on Principles, Devices, and Clinical Applications,” Journal of Clinical Medicine 9 (2020): 460, 10.3390/jcm9020460. 32046072 PMC7073974

[49] A. De Maio , G. Alfieri , M. Mattone , P. Ghanouni , and A. Napoli , “High‐intensity Focused Ultrasound Surgery for Tumor Ablation: A Review of Current Applications.” Radiology‐Imaging Cancer 6 (2024): e230074.. 38099828 10.1148/rycan.230074 PMC10825716

[50] N. Frazier , A. Payne , J. de Bever , et al., “High Intensity Focused Ultrasound Hyperthermia for Enhanced Macromolecular Delivery,” Journal of Controlled Release 241 (2016): 186–193, 10.1016/j.jconrel.2016.09.030. 27686583

[51] H. Ashar and A. Ranjan , “Immunomodulation and Targeted Drug Delivery With High Intensity Focused Ultrasound (HIFU): Principles and Mechanisms,” Pharmacology & Therapeutics 244 (2023): 108393, 10.1016/j.pharmthera.2023.108393. 36965581 PMC12323623

[52] D. Baeza Moyano , D. Arranz Paraiso , and R. Alonso Gonzalez‐Lezcano , “Possible Effects on Health of Ultrasound Exposure, Risk Factors in the Work Environment and Occupational Safety Review.” Healthcare 10 (2022): 423. 35326901 10.3390/healthcare10030423 PMC8954895

[53] A. Fomenko , C. Neudorfer , R. F. Dallapiazza , S. K. Kalia , and A. M. Lozano , “Low‐intensity Ultrasound Neuromodulation: An Overview of Mechanisms and Emerging Human Applications,” Brain Stimulation 11 (2018): 1209–1217, 10.1016/j.brs.2018.08.013. 30166265

[54] X. Hu , T. Zeng , C. C. Husic , and M. J. Robb , “Mechanically Triggered Release of Functionally Diverse Molecular Payloads From Masked 2‐Furylcarbinol Derivatives,” ACS Central Science 7 (2021): 1216–1224, 10.1021/acscentsci.1c00460. 34345671 PMC8323246

[55] Y. Sun , W. J. Neary , Z. P. Burke , H. Qian , L. Zhu , and J. S. Moore , “Mechanically Triggered Carbon Monoxide Release With Turn‐On Aggregation‐Induced Emission,” Journal of the American Chemical Society 144 (2022): 1125–1129, 10.1021/jacs.1c12108. 35019277

