# Supplementary material for: Tailored Nanogel Network Topology Enables Clinical Ultrasound‐Induced Mechanochemical Activation for In Vivo Therapy
Source: Angew Chem Int Ed Engl. 2026 May 25;65(30):e4591365. doi: 10.1002/anie.4591365 (PMC13382917; doi:10.1002/anie.4591365)
Supplement: Supplementary file 1 — Supporting File: anie72788‐sup‐0001‐SuppMat.docx [file ANIE-65-e4591365-s001.docx]

Supporting Information

Tailored Nanogel Network Topology Enables Clinical Ultrasound-Induced Mechanochemical Activation for *In Vivo* Therapy

Helin Li, Fangyin Song, Xiaoye Hu, Li Jing, Qi Shuai, Weike Su*, Andrij Pich*, Xin Li*

DOI: 10.1002/anie.2016XXXXX

Experimental Procedures

**Materials**

All chemical reagents were of analytical grade and were used without further purification, unless otherwise stated. Azobisisobutyronitrile (AIBN, >98%, Adamas-beta), N,N-diisopropylethylamine (DIPEA, 99%, Adamas-beta), N-hydroxysuccinimide (NHS, 98%, Adamas-beta), 1-ethy1-3-(3-dimethylaminopropyl)carbodiimide hydrochloride (EDC⋅HCl, >97%, Adamas-beta), sodium hydroxide (NaOH, >98%, Adamas-beta), succinic anhydride (99%, Adamas-beta), 4-(dimethylamino)pyridine (DMAP, 99%, Adamas-beta), 2,2'-azobis[2-(2-imidazolin-2-yl)propane] dihydrochloride (AIPH, >98%, Adamas-beta), linear amino-terminated methoxy poly(ethylene glycol) (mPEG-NH_2_) with a molecular weight of 20 kDa or 40 kDa (mPEG_10K_-NH_2_ or mPEG_20K_-NH_2_ or mPEG_40K_-NH_2_, Adamas-beta), 4,4'-Azobis (4-cyanovaleric acid) (ACVA, >98%, Macklin), trifluoroacetic acid (TFA, 99.5%, Macklin), 4-cyano-4-(thiobenzoylthio) pentanoic acid (CTA, >97%, Aladdin) and 2,2'-azinobis(3-ethylbenzothiazoline-6-sulfonic acid) diammonium salt (ABTS, 98%, Meryer) were used as received. Pentafluorophenyl methacrylate (PFPMA, 98%, Macklin) and oligo (ethylene glycol) methacrylate (OEGMA, Mw = 450 Da, Bidepharm) were purified from inhibitor by passing them through a column of basic alumina before use.

**Synthesis of super-low molecular weight brush polymers (SLB polymers)**

***Synthesis of poly-PFPMA:*** PFPMA (1 g, 4.202 mmol), AIBN (9 mg, 0.055 mmol), and CTA (123 mg, 0.44 mmol) were dissolved in 1,4-dioxane (3.5 mL) in a Schlenk flask. The solution was subjected to three freeze-pump-thaw cycles whereupon the sealed flask was immersed in a preheated oil bath at 75 °C for 4 h. The reaction was stopped by immersing the reaction flask in cold water. After removal of the solvent in vacuo, the mixture was dissolved in dichloromethane (DCM) and precipitated in hexane. The precipitation was repeated three times by dissolving the residue in little DCM to afford poly-PFPMA. The molecular weight of poly-PFPMA was determined by ^19^F-NMR spectrum using TFA as an internal standard.

**Figure S1.** Synthetic route of poly-PFPMA.


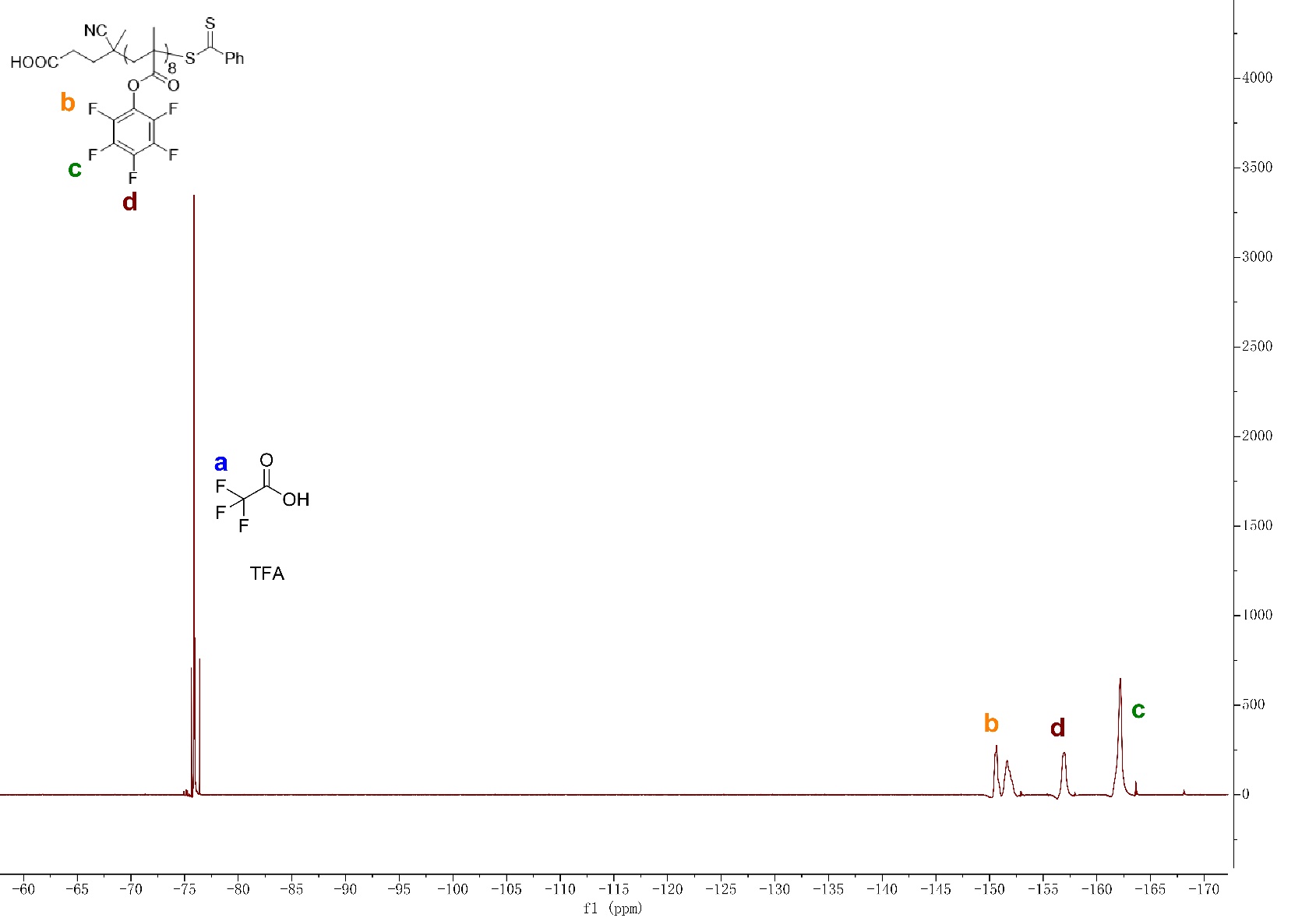


**Figure S2.** ^19^F-NMR spectrum of poly-PFPMA.

***Synthesis of polymers P1:*** OEGMA (563 mg, 1.25 mmol), AIBN (3 mg, 0.018 mmol), and poly-PFPMA (100 mg, 0.05 mmol) were dissolved in 1,4-dioxane (2.5 mL) in a Schlenk flask. The solution was subjected to three freeze-pump-thaw cycles whereupon the sealed flask was immersed in a preheated oil bath at 75 °C for 4 h. The reaction was stopped by immersing the reaction flask in cold water. After removal of the solvent in vacuo, the mixture was dissolved in DCM and precipitated in hexane. The precipitation was repeated three times by dissolving the residue in little DCM to afford P1.

^1^H NMR spectra of all copolymers were collected on an NMR spectroscopy (Bruker Avance NEO 400, Bruker, German). The molecular weight of all copolymers was determined using gel permeation chromatography (GPC, Waters 1515-2414, Waters, USA).

**Figure S3.** Synthetic route of P1.


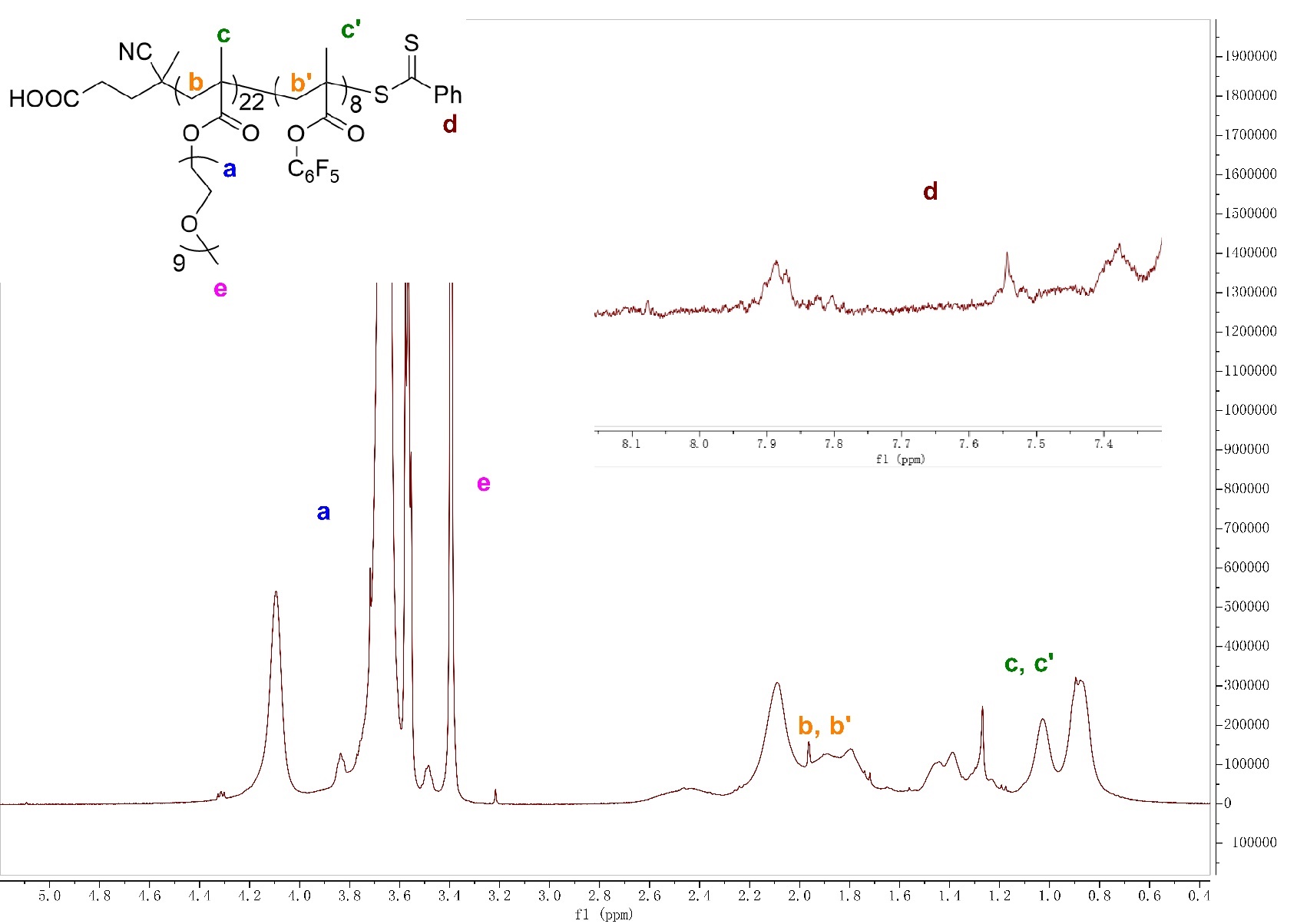


**Figure S4.** ^1^H-NMR spectrum of P1.





**Figure S5.** GPC trace of P1 (Mn = 12 kDa, PDI = 1.19).

***Synthesis of SLB polymers:*** P1 (200 mg, 16.7 µmol) and ACVA (93 mg, 0.333 mmol) were dissolved in the mixture of anhydrous 1,4-dioxane/dimethyl sulfoxide (4 mL, v:v = 4:1). The mixture was stirred and heated at 80 °C for 2 h. The copolymer was precipitated in ether (100 mL) for three times. The crude product was dried under vacuum to afford SLB polymers.

**Figure S6.** Synthetic route of SLB polymers.


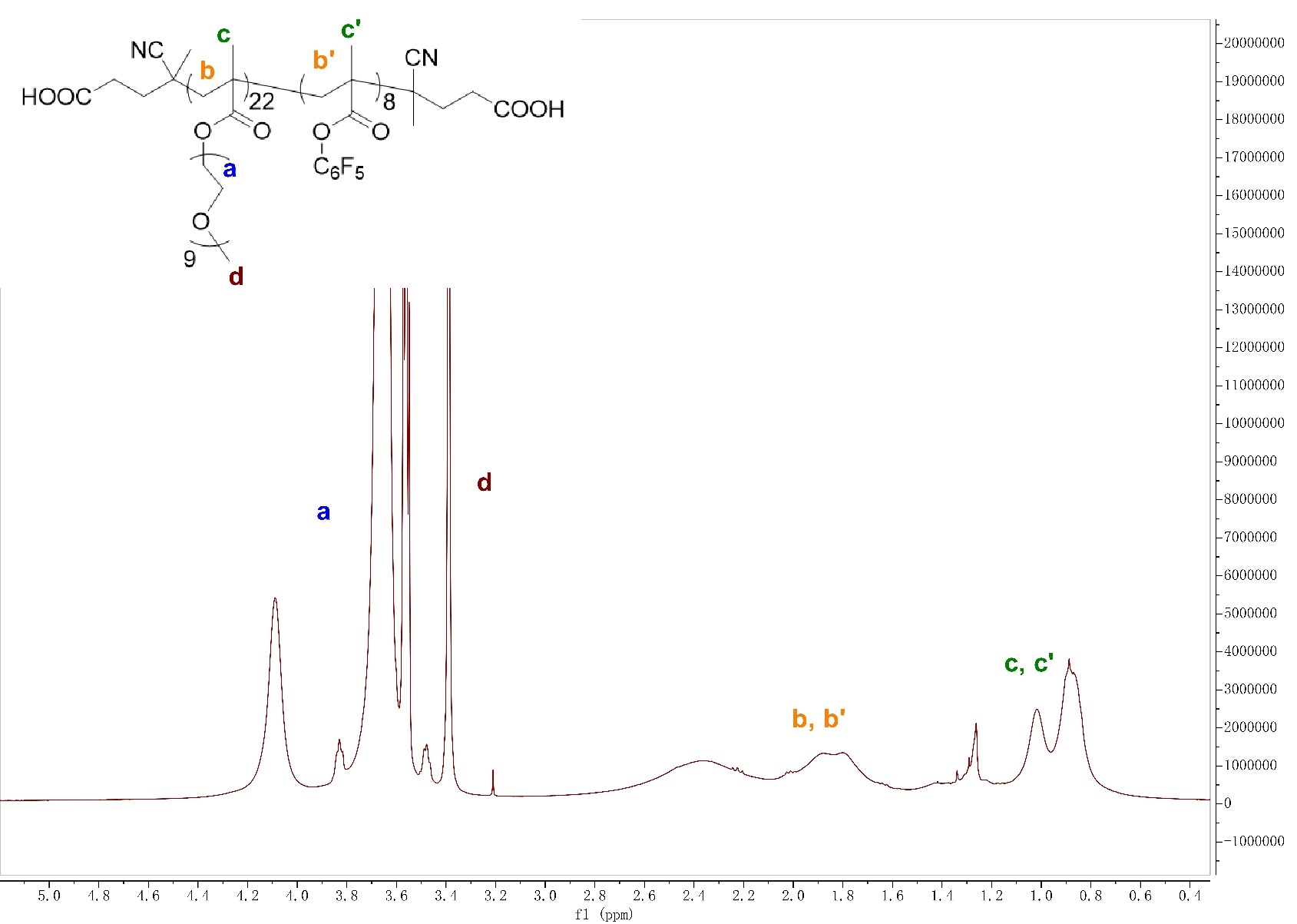


**Figure S7.** ^1^H-NMR spectrum of SLB polymers.





**Figure S8.** GPC trace of SLB polymers (Mn = 12 kDa, PDI = 1.21).

**Synthesis of low molecular weight brush polymers (LB polymers)**

***Synthesis of P2:*** OEGMA (2.925 g, 6.5 mmol), AIBN (5 mg, 0.03 mmol), and poly-PFPMA (250 mg, 0.125 mmol) were dissolved in 1,4-dioxane (8 mL) in a Schlenk flask. The solution was subjected to three freeze-pump-thaw cycles whereupon the sealed flask was immersed in a preheated oil bath at 75 °C for 4 h. The reaction was stopped by immersing the reaction flask in cold water. After removal of the solvent in vacuo, the mixture was dissolved in DCM and precipitated in hexane. The precipitation was repeated three times by dissolving the residue in little DCM to afford P2.

**Figure S9.** Synthetic route of P2.


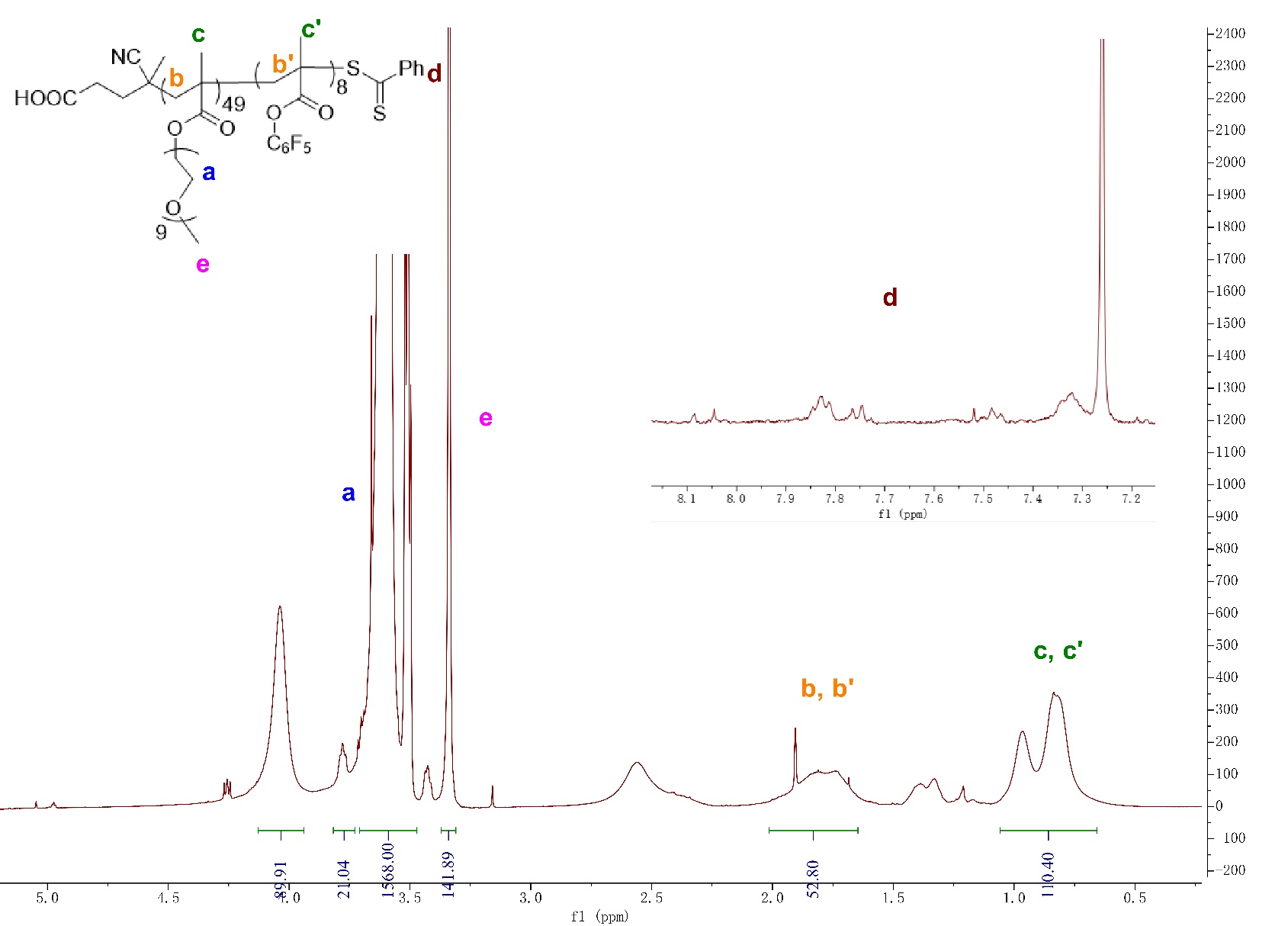


**Figure S10.** ^1^H-NMR spectrum of P2.





**Figure S11.** GPC trace of P2 (Mn = 24 kDa, PDI = 1.18).

***Synthesis of LB polymers:*** P2 (150 mg, 6.25 µmol) and ACVA (35 mg, 0.125 mmol) were dissolved in the mixture of anhydrous 1,4-dioxane/dimethyl sulfoxide (7.5 mL, v:v = 4:1). The mixture was stirred and heated at 80 °C for 2 h. The copolymer was precipitated in ether (100 mL) for three times. The crude product was dried under vacuum to afford LB polymers.

**Figure S12.** Synthetic route of LB polymers.


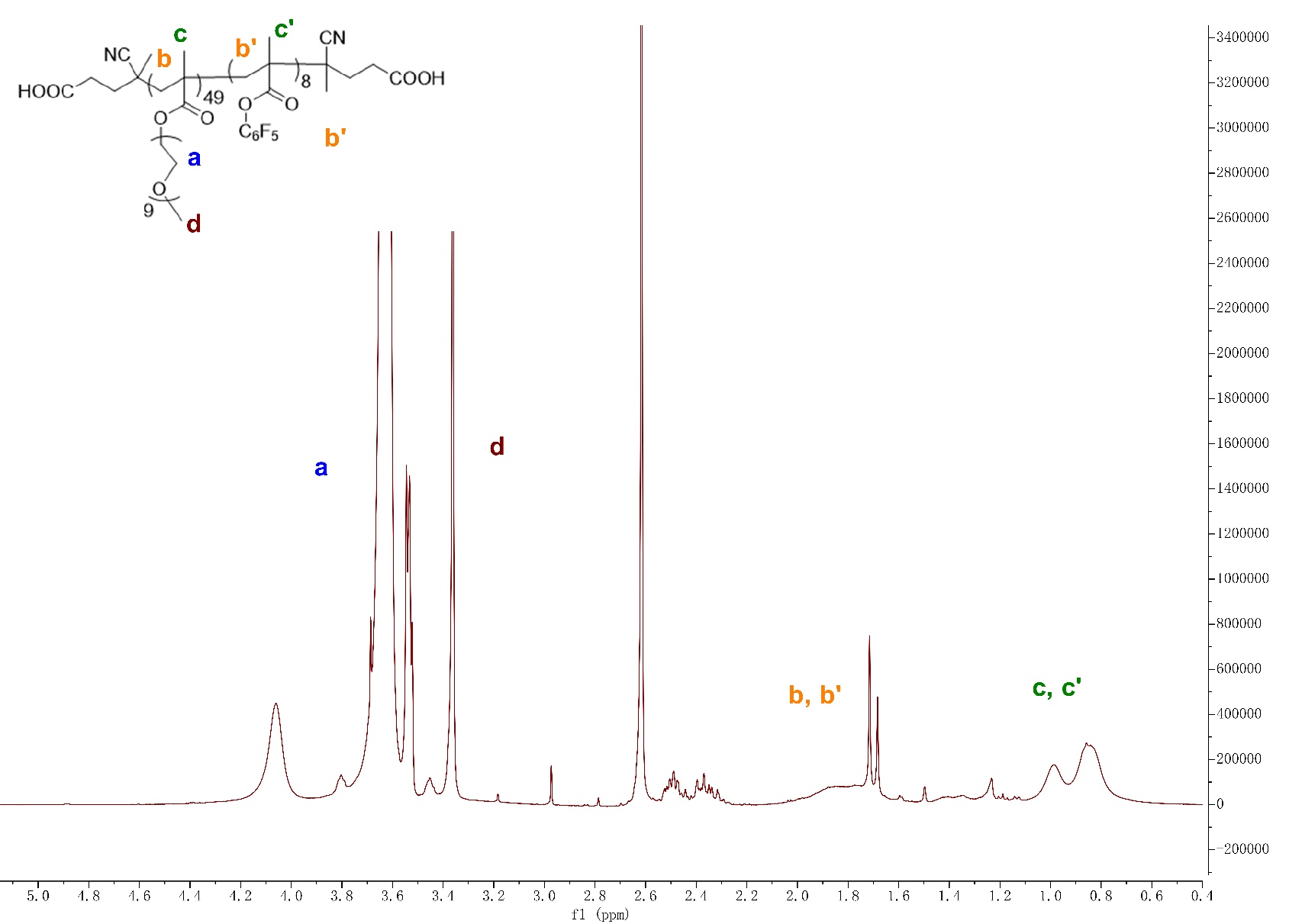


**Figure S13.** ^1^H-NMR spectrum of LB polymers.





**Figure S14.** GPC trace of LB polymers (Mn = 24 kDa, PDI = 1.16).

**Synthesis of high molecular weight brush polymers (HB polymers)**

***Synthesis of polymers P3:*** OEGMA (3.848 g, 8.551 mmol), AIBN (3 mg, 0.018 mmol), and poly-PFPMA (150 mg, 0.075 mmol) were dissolved in 1,4-dioxane (8 mL) in a Schlenk flask. The solution was subjected to three freeze-pump-thaw cycles whereupon the sealed flask was immersed in a preheated oil bath at 75 °C for 4 h. The reaction was stopped by immersing the reaction flask in cold water. After removal of the solvent in vacuo, the mixture was dissolved in DCM and precipitated in hexane. The precipitation was repeated three times by dissolving the residue in little DCM to afford P3.

**Figure S15.** Synthetic route of P3.


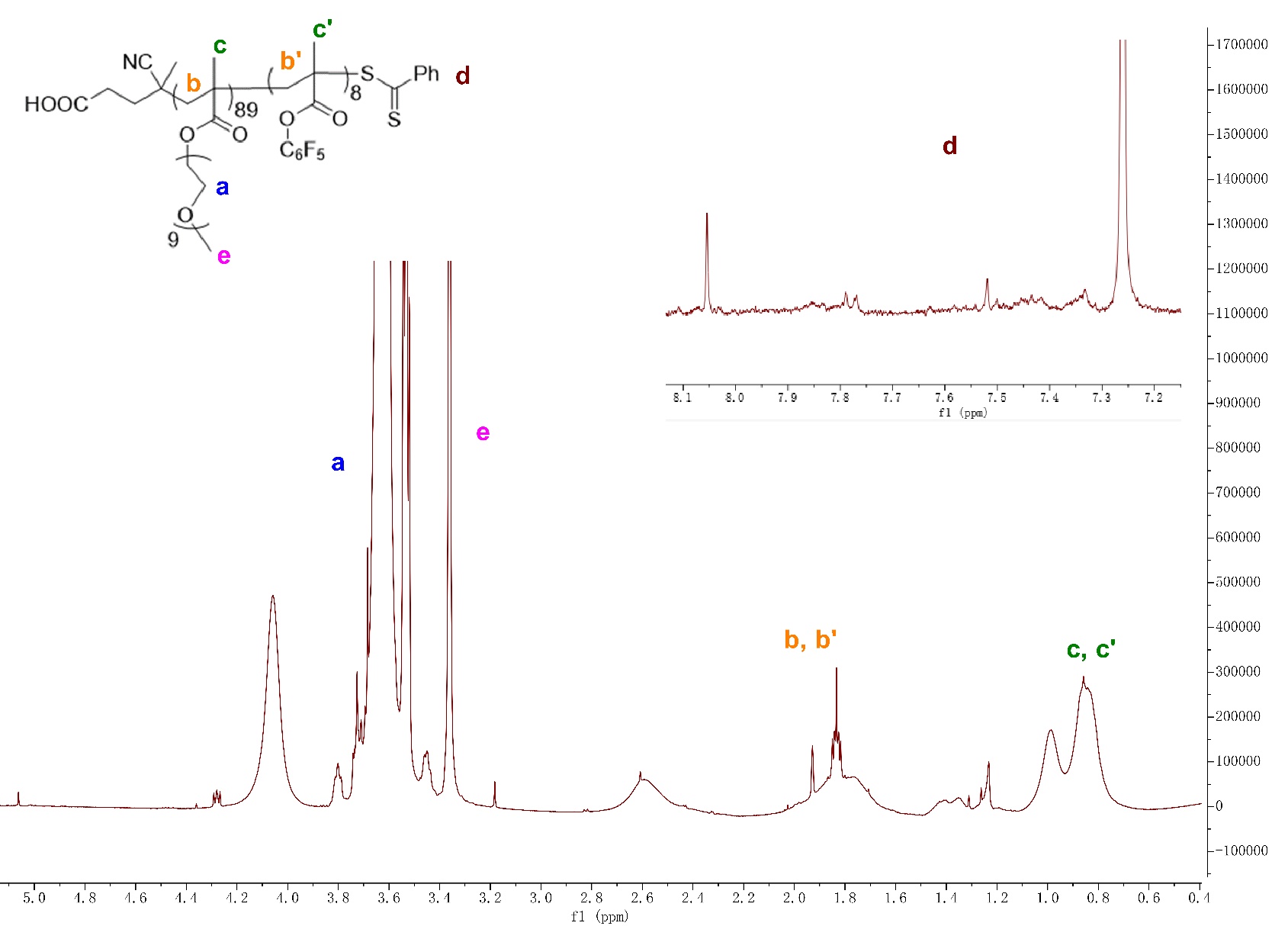


**Figure S16.** ^1^H-NMR spectrum of P3.





**Figure S17.** GPC trace of P3 (Mn = 42 kDa, PDI=1.25).

***Synthesis of HB polymers:*** P3 (250 mg, 0.059 mmol) and ACVA (333 mg, 1.189 mmol) were dissolved in a mixture of anhydrous 1,4-dioxane/dimethyl sulfoxide (25 mL, v:v = 4:1). The mixture was stirred and heated at 80 °C for 2 h. The copolymer was precipitated in ether (100 mL) for three times. The crude product was dried under vacuum to afford HB polymers.

**Figure S18.** Synthetic route of HB polymers.


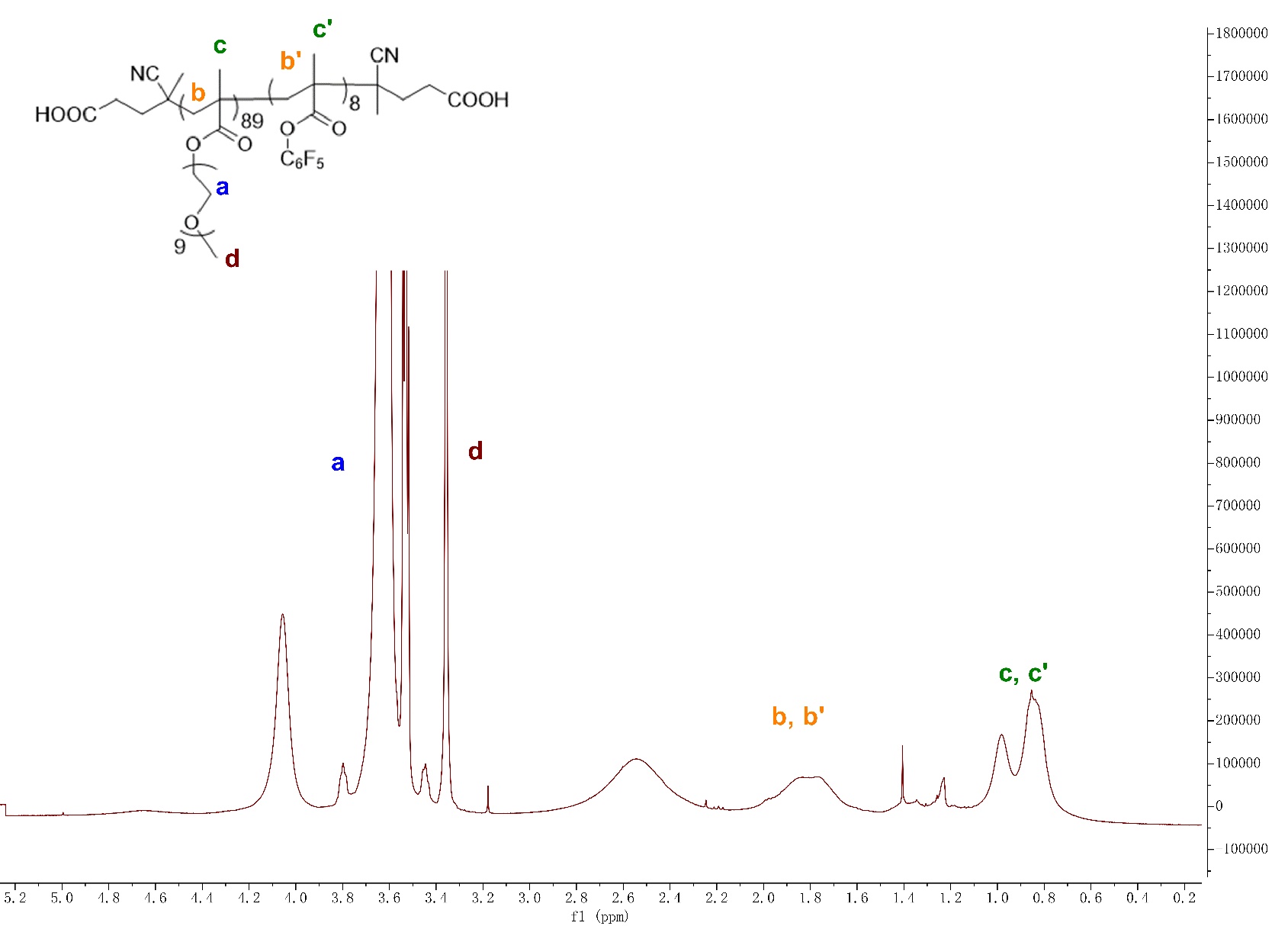


**Figure S19.** ^1^H-NMR spectrum of HB polymers.





**Figure S20.** GPC trace of HB polymers (Mn = 42 kDa, PDI=1.12).

**Synthesis of super-low molecular weight single branched polymers (SLS polymers)**

***Synthesis of P4:*** poly-PFPMA (200 mg, 0.1 mmol) was dissolved in DCM (10 mL), and then mPEG_10k_-NH_2_ (100 mg, 0.01 mmol) and DIPEA (7 μL, 0.04 mmol) was added. The mixture was stirred for 48 h at room temperature. After removal of the solvent in vacuo, the mixture was dissolved in little DCM and precipitated in hexane. The precipitation was washed three times with cold tetrahydrofuran (THF) and dried to obtain P4.

**Figure S21.** Synthetic route of P4.


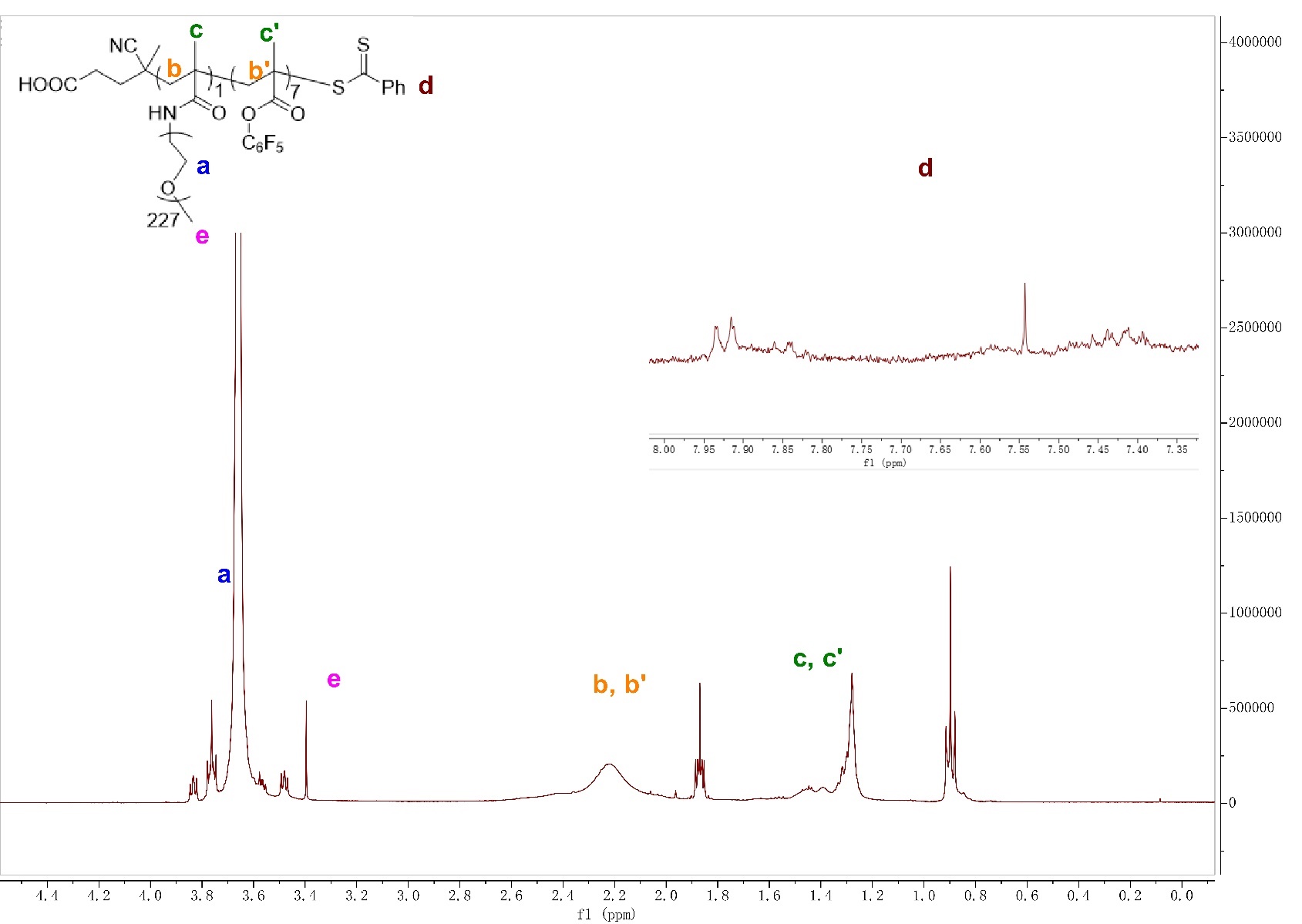


**Figure S22.** ^1^H-NMR spectrum of P4.





**Figure S23.** GPC trace of P4 polymer (Mn = 12 kDa, PDI = 1.25).

**Figure S24.** Synthetic route of SLS polymers.


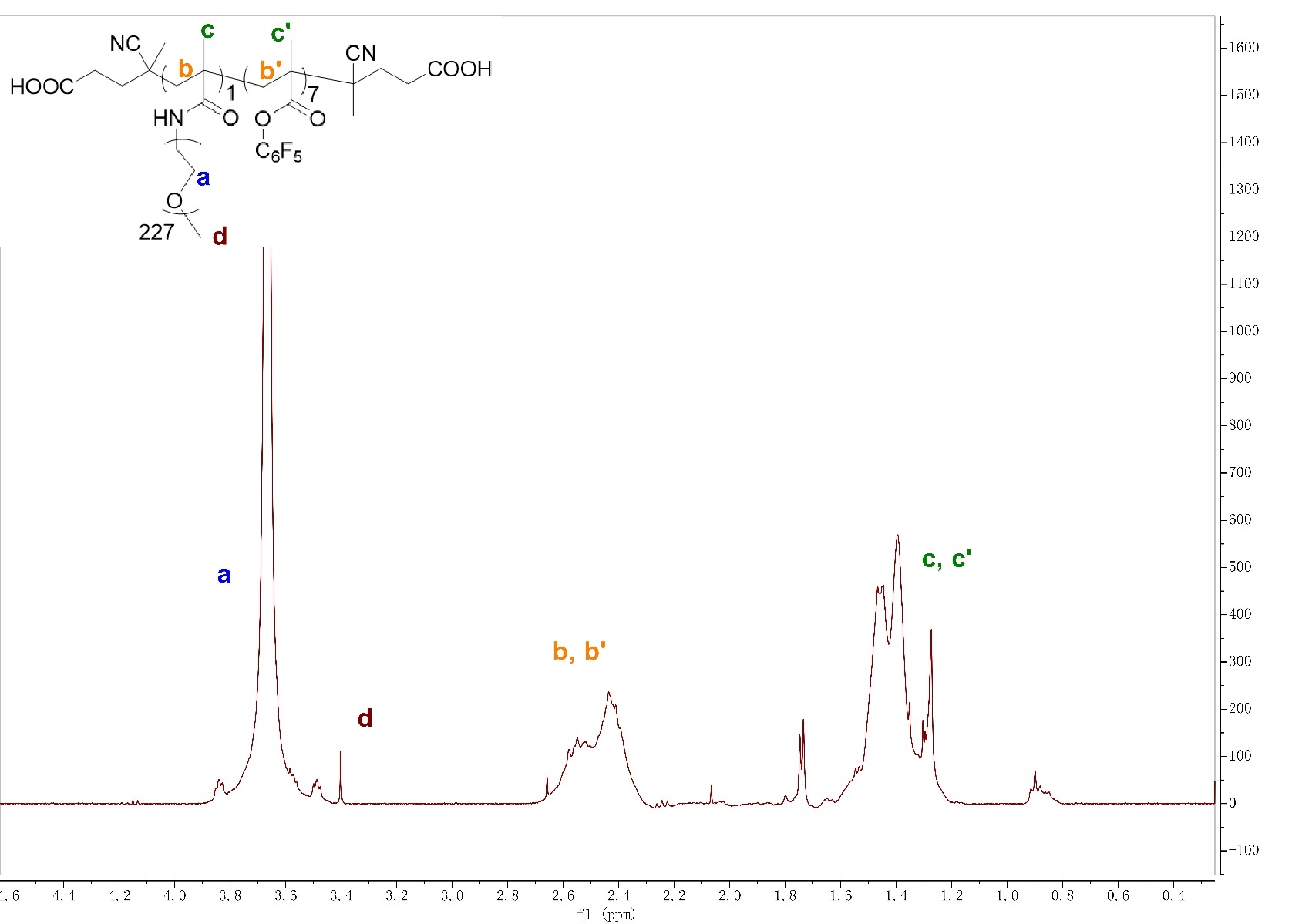


**Figure S25.** ^1^H-NMR spectrum of SLS polymer.





**Figure S26.** GPC trace of SLS polymer (Mn = 12 kDa, PDI = 1.21).

**Synthesis of low molecular weight single branched polymers (LS polymers)**

***Synthesis of P5:*** poly-PFPMA (200 mg, 0.1 mmol) was dissolved in DCM (10 mL), and then mPEG_20k_-NH_2_ (200 mg, 0.01 mmol) and DIPEA (7 μL, 0.04 mmol) was added. The mixture was stirred for 48 h at room temperature. After removal of the solvent in vacuo, the mixture was dissolved in little DCM and precipitated in hexane. The precipitation was washed three times with cold THF and dried to obtain P3.

**Figure S27.** Synthetic route of P5.


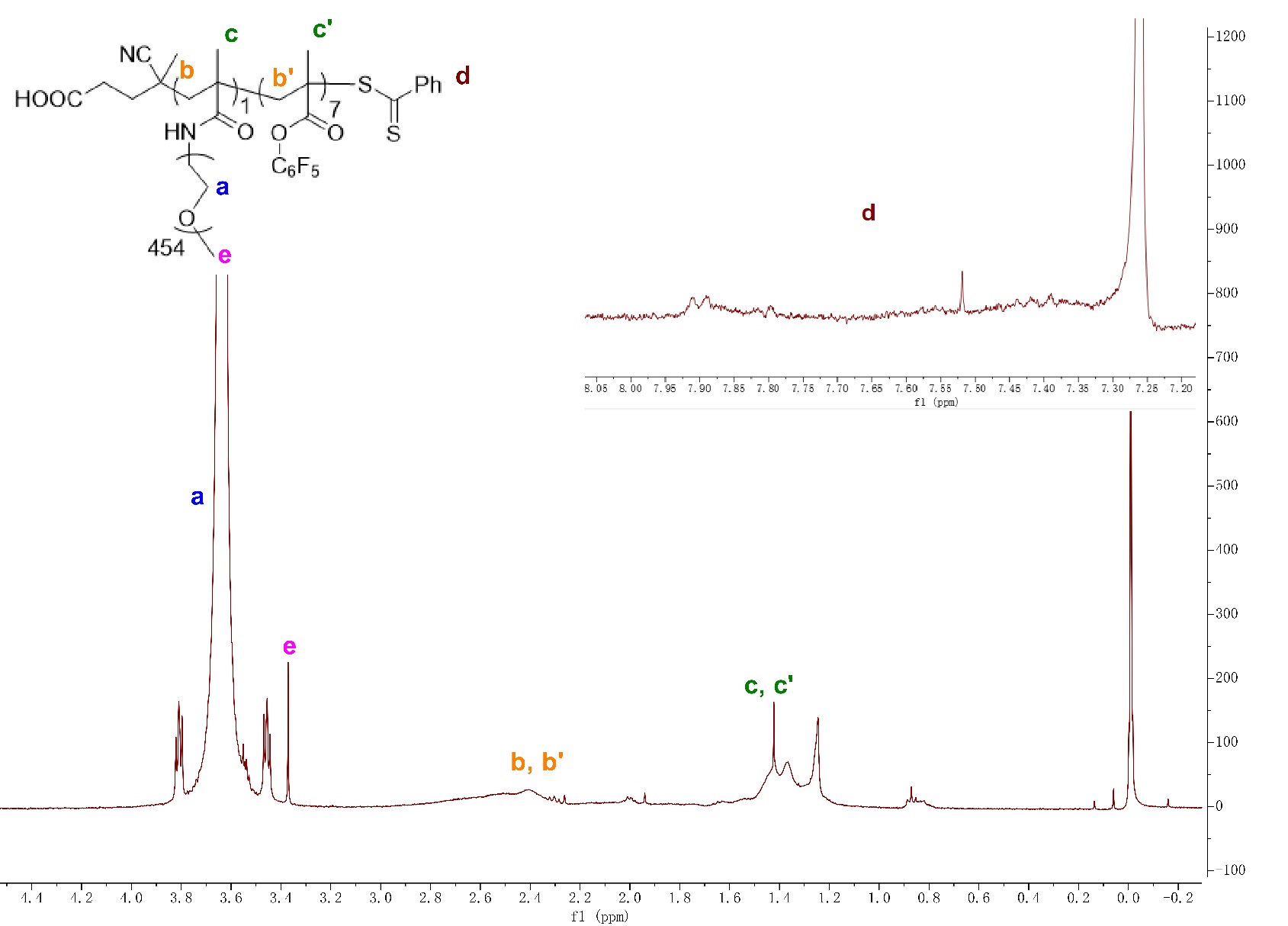


**Figure S28.** ^1^H-NMR spectrum of P5.





**Figure S29.** GPC trace of P5 (Mn = 22 kDa, PDI=1.02).

***Synthesis of LS polymers:*** P5 (220 mg, 0.01 mmol) and ACVA (56 mg, 0.2 mmol) were dissolved in a mixture of anhydrous 1,4-dioxane/dimethyl sulfoxide (7.5 mL, v:v = 4:1). The mixture was stirred and heated at 80 °C for 2 h. The copolymer was precipitated in ether (100 mL) for three times. The crude product was dried under vacuum to afford LS polymers.

**Figure S30.** Synthetic route of LS polymers.


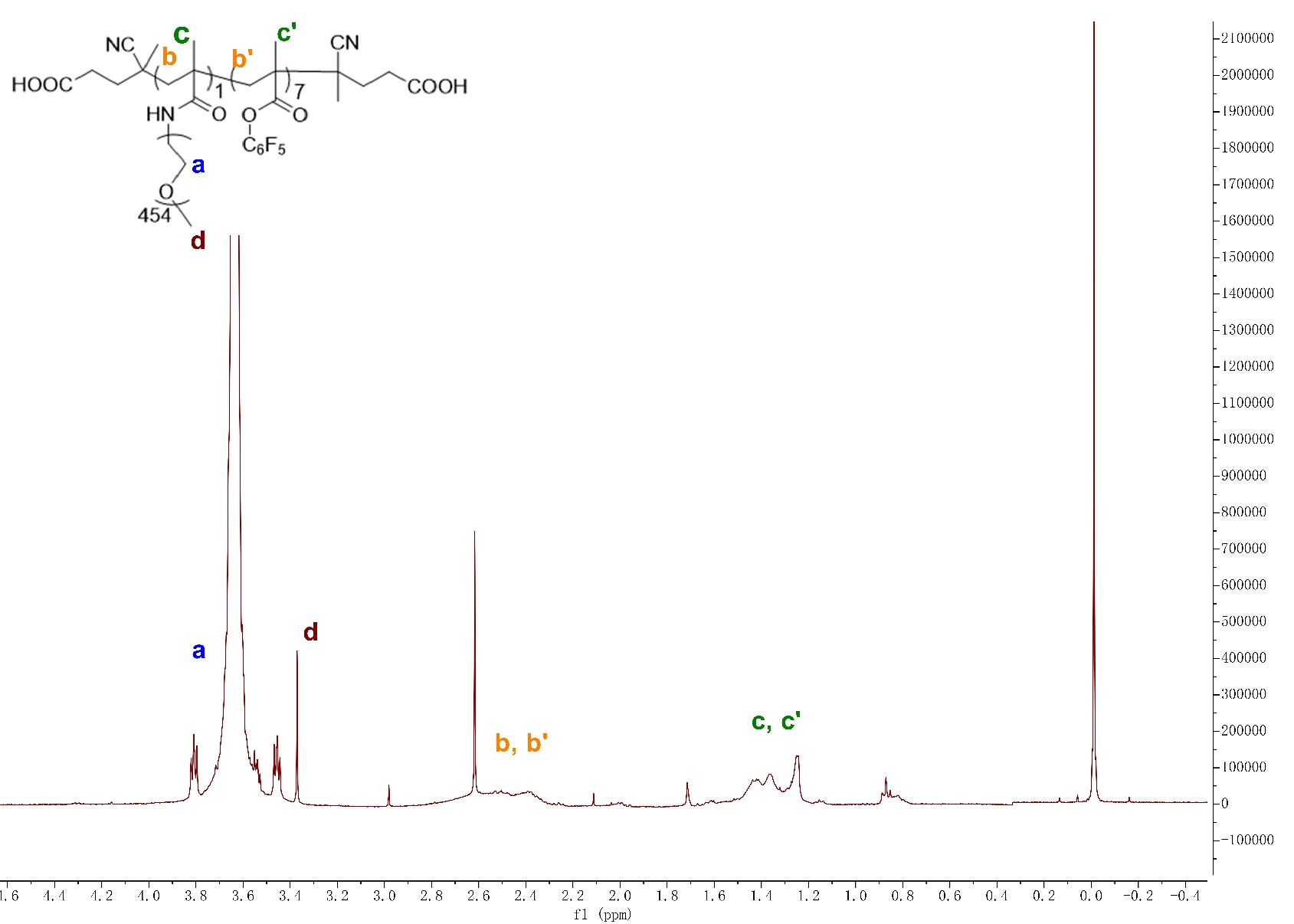


**Figure S31.** ^1^H-NMR spectrum of LS polymers.





**Figure S32.** GPC trace of LS polymers (Mn = 22 kDa, PDI=1.02).

**Synthesis of high molecular weight single branched polymers (HS polymers)**

***Synthesis of P6:*** poly-PFPMA (200 mg, 0.1mmol) was dissolved in DCM (20 mL), and then mPEG_40k_-NH_2_ (400 mg, 0.01mmol) and DIPEA (7 μL, 0.04 mmol) was added. The mixture was stirred for 48 h at room temperature. After removal of the solvent in vacuo, the mixture was dissolved in little DCM and precipitated in hexane. The precipitation was washed three times with cold THF and dried to obtain P6.

**Figure S33.** Synthetic route of P6.


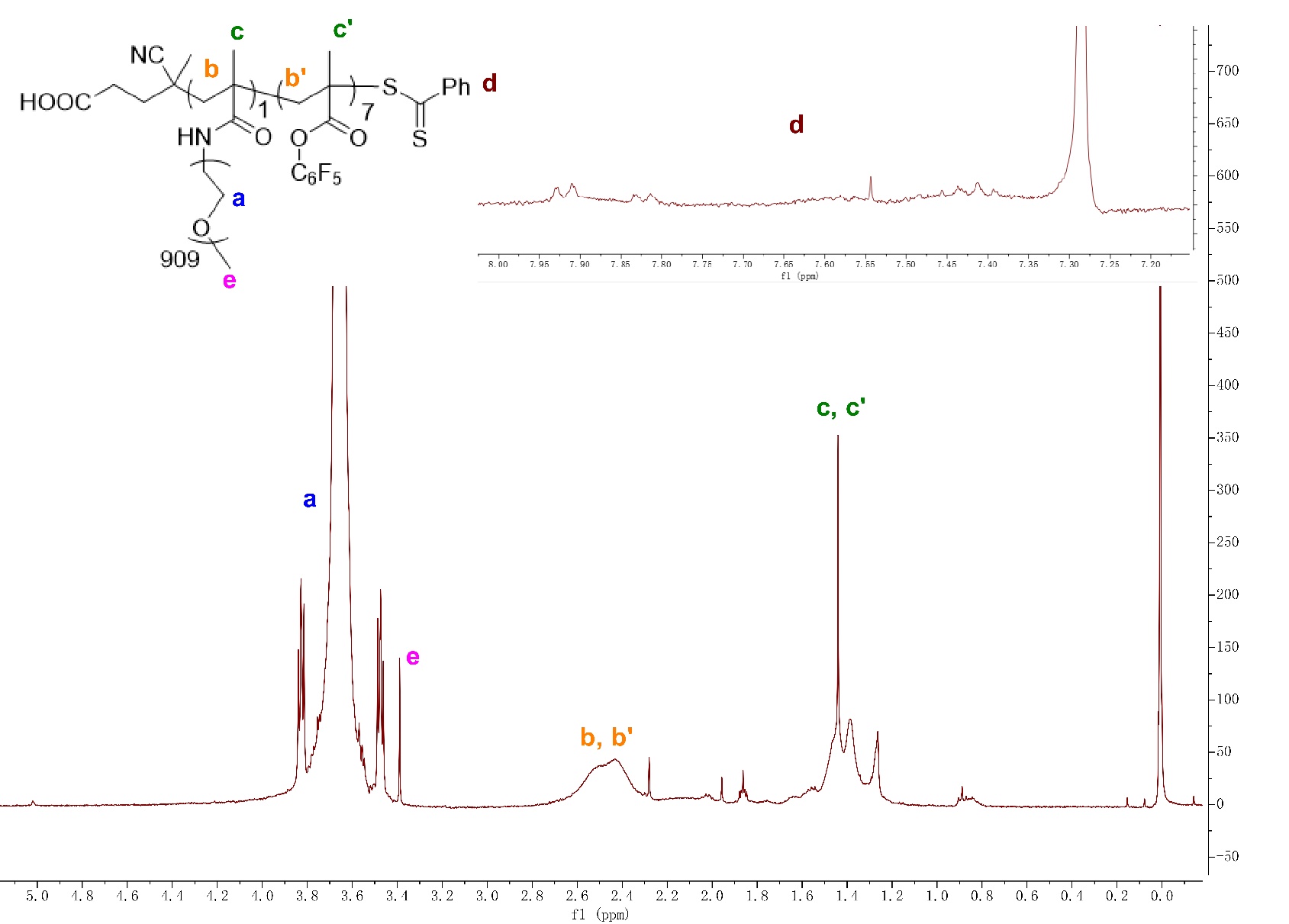


**Figure S34.** ^1^H-NMR spectrum of P6.





**Figure S35.** GPC trace of P6 (Mn = 42 kDa, PDI=1.02)

***Synthesis of HS polymers:*** P6 (420 mg, 0.01 mmol) and ACVA (56 mg, 0.2 mmol) were dissolved in mixture of anhydrous 1,4-dioxane/dimethyl sulfoxide (7.5 mL, v:v = 4:1). The mixture was stirred and heated at 80 °C for 2 h. The copolymer was precipitated in ether (100 mL) for three times. The crude product was dried under vacuum to afford HS polymers.

**Figure S36.** Synthetic route of HS polymers.


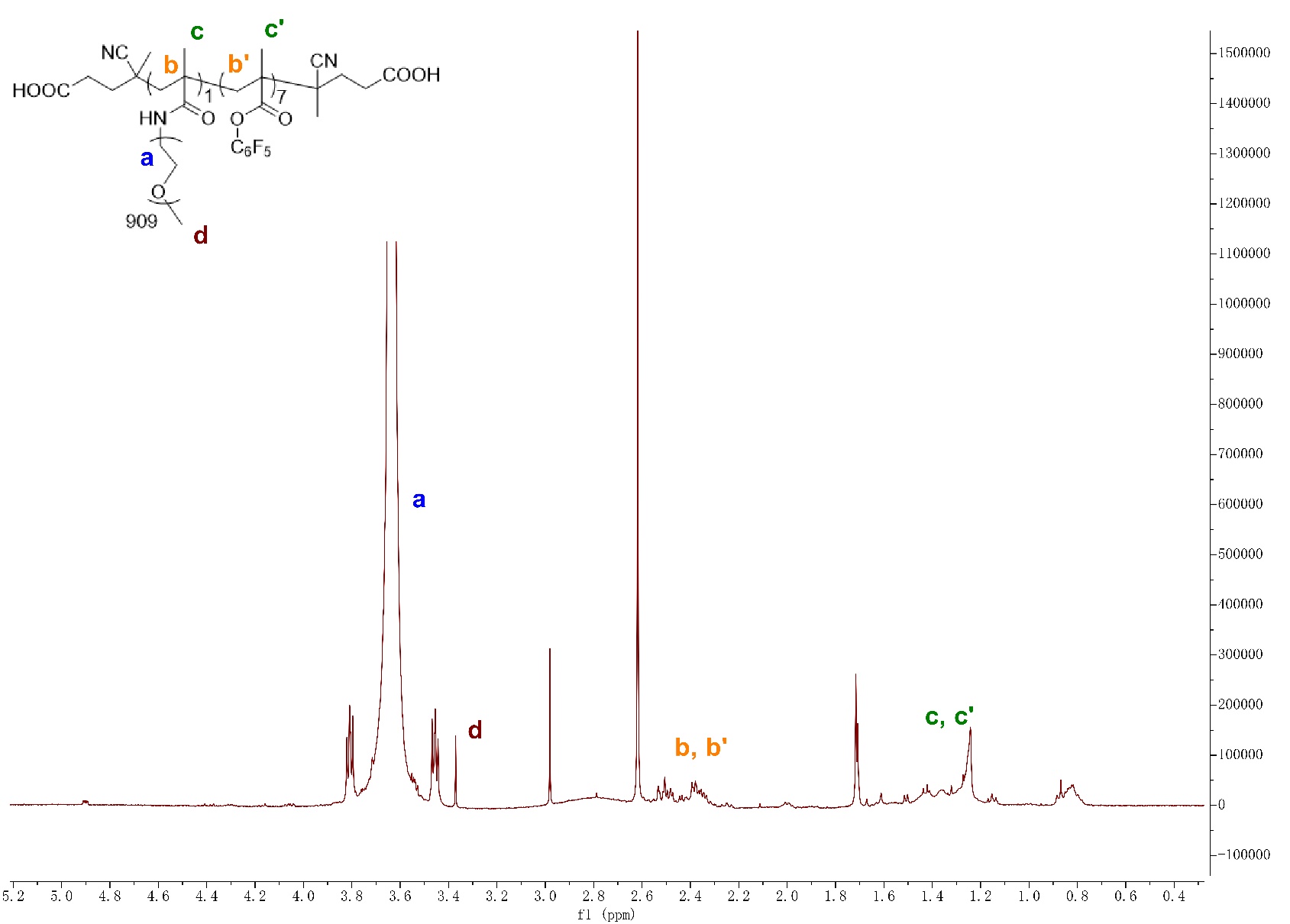


**Figure S37.** ^1^H-NMR spectrum of HS polymers.





**Figure S38.** GPC trace of HS polymers (Mn = 42 kDa, PDI=1.02).

**Synthesis of NGs with different network topologies**

SLB polymers (50 mg, 0.0042 mmol), LB polymers (100 mg, 0.0042 mmol), HB polymers (175 mg, 0.0042 mmol), SLS polymers (50 mg, 0.0042 mmol), LS polymers (92 mg, 0.0042 mmol), and HS polymers (175 mg, 0.0042 mmol) were dissolved in dry THF (10 mg/mL) respectively, and then AIPH aqueous solution (0.336 mL, 0.0052 mmol) and DIPEA (6 μL, 0.033 mmol) were added. The mixture was stirred for 48 h at room temperature to obtain LB NGs, HB NGs, LS NGs, and HS NGs, respectively. Afterward, these six NGs were dialyzed against water using dialysis bag (MWCO = 8000-12000 Da) for 3 d, respectively. The morphology of NGs was characterized using transmission electron microscope (TEM, HT7700, Hitachi, Japan) at an accelerating voltage of 120 KV. The hydrodynamic diameter and polydispersity index (PDI) of NGs were determined using dynamic light scattering (DLS) equipped with a Zetasizer (Nano ZS90, Malvern, UK).

**Figure S39.** Synthetic route of NGs with different network topologies.


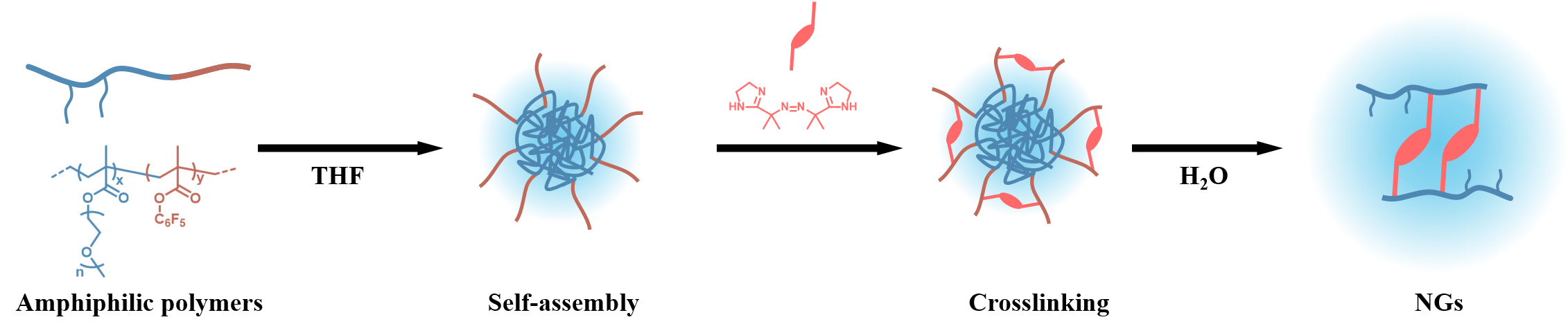


**Figure S40.** Mechanism of NG formation.

**Synthesis of non-crosslinked polymers bearing AIPH (LB-A polymers)**

LB polymers (120 mg, 0.005 mmol) were dissolved in DMSO (10 mg/mL), and then AIPH (32 mg, 0.1 mmol) and DIPEA (17 μL, 0.1 mmol) was added. The mixture was stirred for 48 h at room temperature. The mixture was dialyzed against water using dialysis bag (MWCO = 8000-12000 Da) for 3 d, respectively. The dialyzed aqueous solution was lyophilized to afford LB-A polymers.

**Figure S41.** Synthetic route of LB-A polymers.


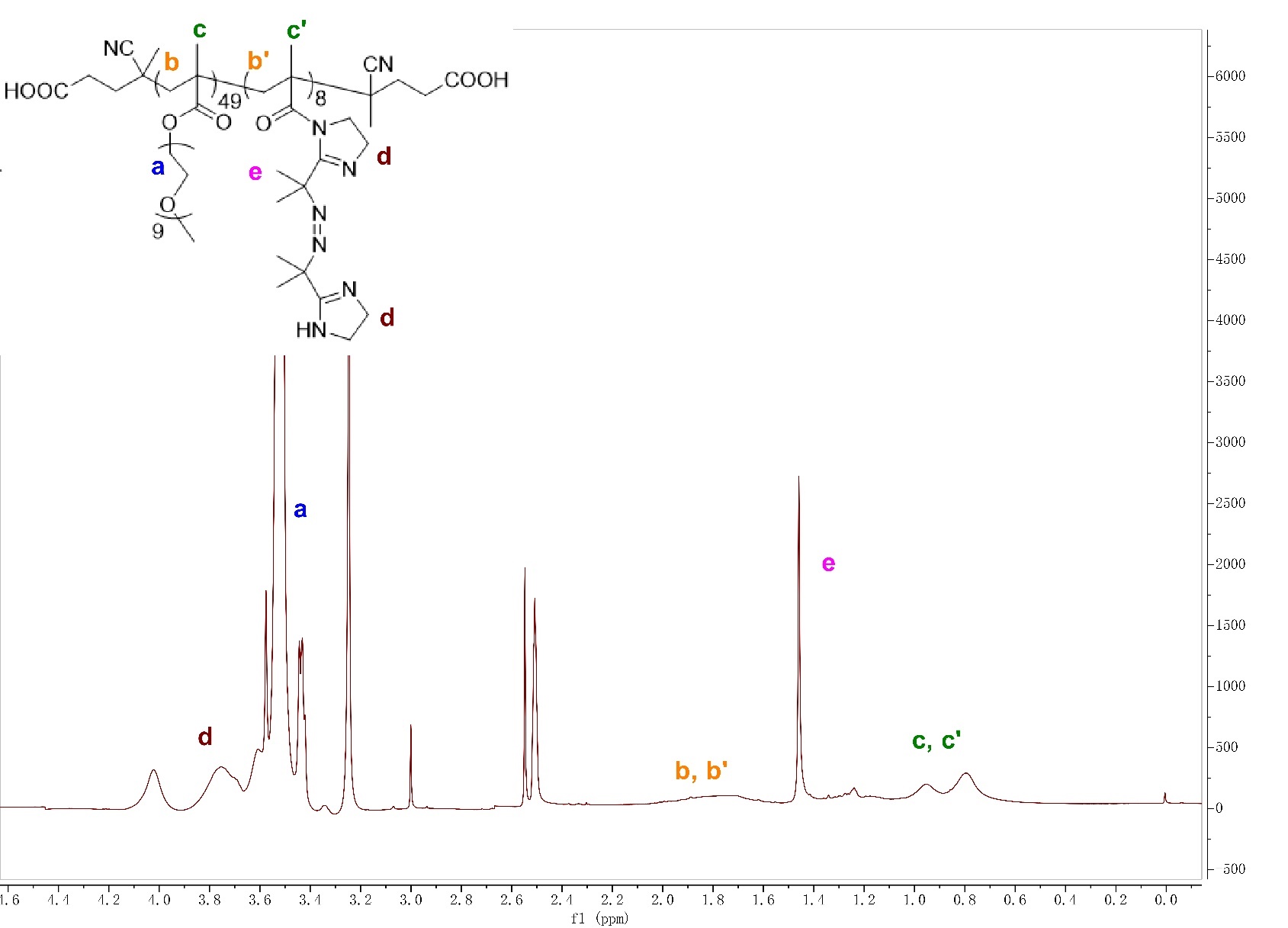


**Figure S42.** ^1^H-NMR spectrum of LB-A polymers.

**Synthesis of PAP**

AIPH (2.6 g, 8.05 mmol) was dissolved in water (30 mL) and NaOH solution (2 M, 10 mL) was added, and then stirred. The precipitation was filtered, washed with water, and the solvent was dried to obtain a white solid 2,2'-azobis[2-(2-imidazolin-2-yl)propane] (AIP). Next, AIP (500 mg, 2 mmol), succinic anhydride (420 mg, 4.2 mmol) and DMAP (73 mg, 0.6 mmol) were dissolved in DCM (50 mL) and stirred for 1 d at room temperature to obtain AIP-COOH. Thin layer chromatography (TLC) was used to monitor the reaction process. AIP-COOH (1.97 mg, 0.005 mmol), EDC (2.88 mg, 0.015 mmol), NHS (1.4 mg, 0.012 mmol), DIPEA (10 μL, 0.08 mmol), and mPEG_20k_-NH_2_ (200 mg, 0.01 mmol) were dissolved in DCM (10 mL), and stirred at room temperature for 1 d. The resulting solution was dialyzed against water using the dialysis bag (MWCO = 20000 Da) for 3 d, and then lyophilized to obtain PAP.

**Figure S43.** Synthetic route of PAP.


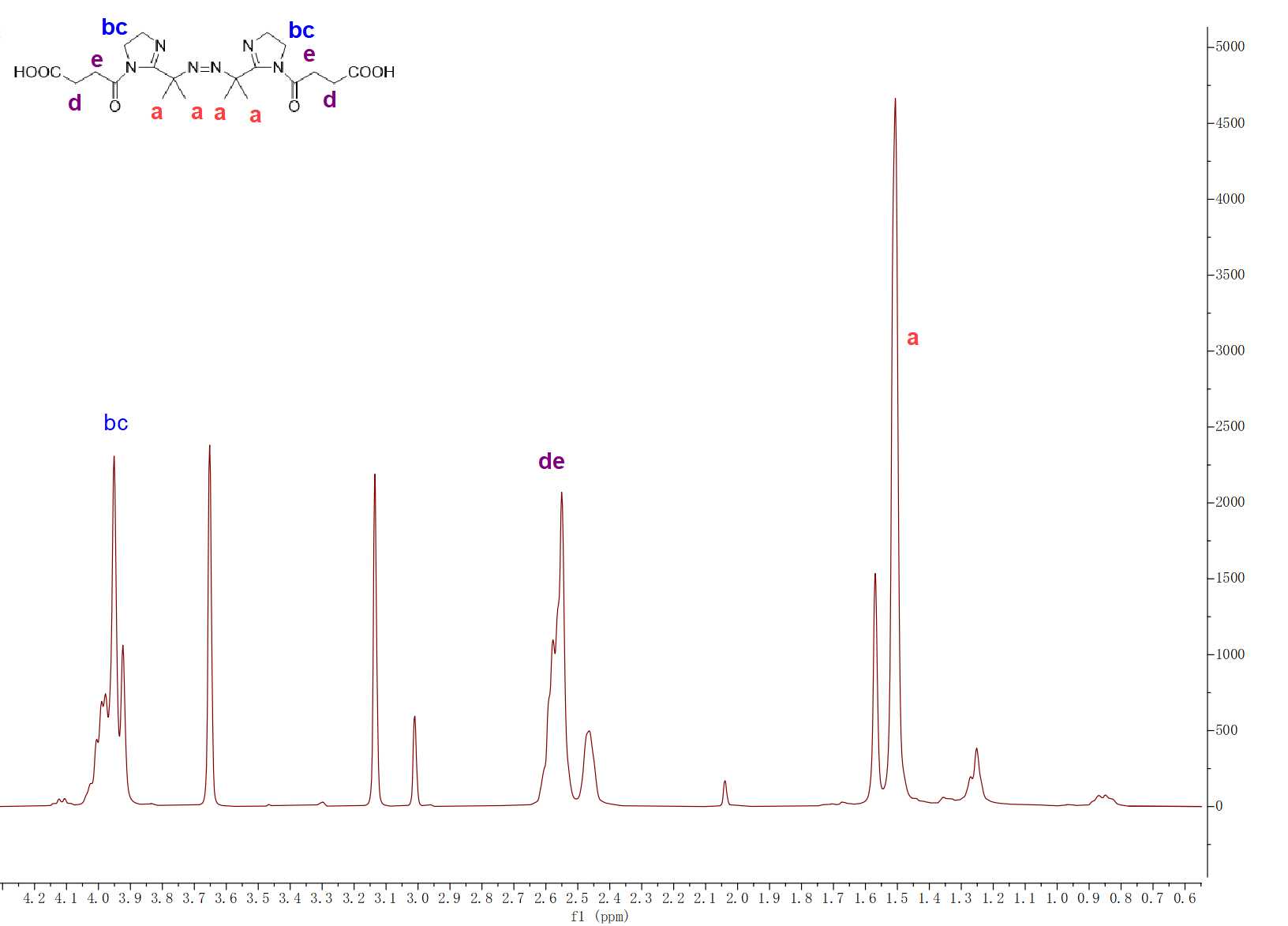


**Figure S44.** ^1^H-NMR spectrum of AIP-COOH.





**Figure S45.** GPC traces of PAP polymers.

**US treatment experiments**

***LFUS treatment:*** The radical generation of NGs upon LFUS by mechanophore activation was examined using ABTS as an indicator. Six NGs containing azo mechanophore (0.238 μmol) were mixed with ABTS (1.5 mg/mL). The mixture (2 mL) was placed into an Eppendorf tube, immersed in an ice-water bath, and then exposed to LFUS using a Sonics VCX 500 W ultrasonic processor (JY92-IIN, Scientz, China) with a 6 mm probe, and 20% of the maximum power of 650 W in pulsed mode (5 s on, 5 s off) at f = 20 kHz for a given time. Following a similar procedure, PAP was treated as a control group. After LFUS treatment, HCl solution (11 μL, 1 M) was added to the mixed solution. The UV-Vis spectra of NGs at 500-900 nm were recorded using UV-Vis spectroscopy (BioTek SYNERGY H1, Agilent, USA). Moreover, during LFUS treatment, the thermal images of the NG solutions were recorded by a handheld thermal imaging instrument (H21 Pro, Hikimicro, China).

***HFUS treatment:*** The radical generation of NGs upon HFUS by mechanophore activation was examined using ABTS as an indicator. Six NGs containing azo mechanophore (0.238 μmol) were mixed with ABTS (1.5 mg/mL). The mixture (2 mL) was placed into a PE plastic bag, coated with a medical US coupling agent, and then subjected to HFUS with high intensity (24 W/cm^2^, f = 2.4 MHz) using Mini HIFU 3rd generation (Dongguan Bomei Trading Co., Ltd., China) or HFUS with low intensity (2 W/cm^2^, f = 1.0 MHz) using a dual-mode US generator (UT1021, Dongdixin Technology Co., Ltd., China) for a given time. Following a similar procedure, LB-A and PAP polymers were treated as a control group. After HFUS treatment, HCl solution (11 μL, 1 M) was added to the mixed solution. The UV-Vis spectra of NGs at 500-900 nm were recorded using UV-Vis spectroscopy. Moreover, during HFUS treatment, the thermal images of the NG solutions were recorded by a handheld thermal imaging instrument.

***Clinical LIFU treatment:*** The radical generation of NGs upon clinical LIFU by mechanophore activation was examined using ABTS as an indicator. Six NGs containing azo mechanophore (0.238 μmol) were mixed with ABTS (1.5 mg/mL). The mixture (2 mL) was put into a sealed PE plastic bag, and placed in a degassed water tank at room temperature. The brightness-mode (B-mode) US was used to track the sample position, and then the system was exposed to LIFU (50 W) in pulsed mode (9990 ms on, 990 ms off) at f = 1.1 MHz using a focused ultrasound therapy system (FEP-BY02, YDME, China) for a given time. Following a similar procedure, PAP was treated as a control group. Afterwards, the influence of the thermal effect of LIFU on the mechanophore activation was investigated. LB NGs mixed with ABTS (1.5 mg/mL) were put into a sealed PE plastic bag, and placed in a degassed water tank at 55 °C. The B-mode US was used to track the sample position, and then the system was exposed to LIFU for a given time. After LIFU treatment, HCl solution (11 μL, 1 M) was added to the mixed solution. The UV-Vis spectra of NGs at 500-900 nm were recorded using UV-Vis spectroscopy.

A standard curve was obtained for the calculation of the activation efficiency of mechanophores as shown in **Figure S50**. Before and after LFUS treatment, the ratio of peak area of PAP was measured using GPC. According to the standard curve, the activation efficiency of mechanophores was estimated by equation (1). Furthermore, the activation rate of mechanophores was estimated by equation (2).

Activation efficiency of mechanophores = (Mn / Mo)×100% (1)

Activation rate of mechanophores = Activation efficiency / US time (2)

where Mn and Mo represent the molar amount of activated azo mechanophore and the total amount of azo mechanophore, respectively.

**Degradation of PAP and NGs after the treatment of LFUS or HFUS**

The solution of PAP (5 mg/mL) was exposed to LFUS, and the molecular weight of PAP before and after US treatment was determined by GPC. Additionally, the solution of HB NGs (10 mg/mL) or LB NGs (10 mg/mL) was exposed to LFUS or HFUS for 5 min, respectively. Before and after US treatment, the morphology of HB NGs or LB NGs was observed using DLS measurement and TEM imaging.

**Thermal stability of NGs**

The NG solution mixed with ABTS was incubated in a water bath at 60 °C for 5 min, and the UV-Vis spectra of the mixed solution were recorded.

**Cell culture and antitumor effect *in vivo***

***HFUS-mediated mechanochemical therapy:*** All animal procedures were approved by the Animal Ethics Committee of Zhejiang University of Technology and performed in accordance with the Guidelines for the Care and Use of Laboratory Animals of Zhejiang University of Technology (No. 20240313010). Murine breast cancer 4T1 cells were cultivated in DMEM supplemented with fetal bovine serum (FBS, 10%), streptomycin (100 μg/mL), and penicillin (100 units/mL). The cells were incubated in a humid atmosphere with 5% CO_2_ at 37 °C. BALB/c female nude mice (4-weeks old) were subcutaneously injected with 1 × 10^6^ 4T1 cells in the right flank on day 5. When the tumors reached about 130 mm^3^, the 4T1 tumor-bearing nude mice were randomly divided into 6 groups (n = 3): (1) PBS group, (2) PAP group, (3) LB NGs group, (4) PBS + HFUS group, (5) PAP + HFUS group, and (6) LB NGs + HFUS group. PAP (100 μL, 10 μg/μL) or LB NGs (50 μL, 10 μg /μL) was intratumorally injected into the mice on day 0, 2 and 4 for one time daily. After injection for 1 h, the tumors were exposed to HFUS with high intensity for 5 min twice daily. The interval between HFUS treatment is 3 min to avoid the temperature increase in tumor. During HFUS treatment, the thermal images of nude mice were recorded by a handheld thermal imaging instrument. The tumor size was recorded every other day, and the tumor volume was calculated via formula V = L/2 × W^2^ after measuring the tumor length (L) and width (W). The tumors were excised on day 0, fixed with 10% formalin, sectioned, and stained with dihydroethidium (DHE). Additionally, the tissues were excised on day 12, and the slides were processed for hematoxylin and eosin (H&E) staining, caspase 1 (Cas-1) staining, caspase 3 (Cas-3) staining, Ki67 staining, and HSP70 staining, respectively.

***Clinical LIFU-mediated synergistic therapy:*** Rat hepatoma N1S1 cells were cultivated in RPMI 1640 supplemented with FBS (10%), streptomycin (100 μg/mL), and penicillin (100 units/mL). The cells were incubated in a humid atmosphere with 5% CO_2_ at 37 °C. Sprague-Dawley (SD) rats (~ 200g) were subcutaneously injected with 2×10^7^ cells in the right flank on day 10. When the tumors reached about 1500 mm^3^, the N1S1 tumor-bearing rats were randomly divided into 3 groups (n = 3): (1) PBS group, (2) PBS + LIFU group, and (3) LB NGs + LIFU group. LB NGs (200 μL, 10 μg/μL) were intratumorally injected into rats on day 0. After injection for 1 h, the tumors were exposed to LIFU irradiation for 1 min twice daily. The interval between LIFU treatment is 1 min. During LIFU treatment, the thermal images of nude mice were recorded by a handheld thermal imaging instrument. The size of the tumor was recorded once daily, and the tumor volume was calculated based on formula V = L/2 × W^2^. The tumor tissues were excised on day 4, fixed with 10% formalin, sectioned, and further processed for H&E staining, Ki67 staining, Cas-3 staining, and HSP70 staining, respectively.

**Statistical Analysis**

The data were shown as mean ± standard deviation (SD), and the significance between two groups of the data in this work was analyzed on the basis of one-way ANOVA test. Values of *P < 0.05 and **P < 0.01 and ***P < 0.001 were considered statistically significant.





**Figure S46.** Absolute peak areas of PAP before and after treatment of LFUS for 5 min.


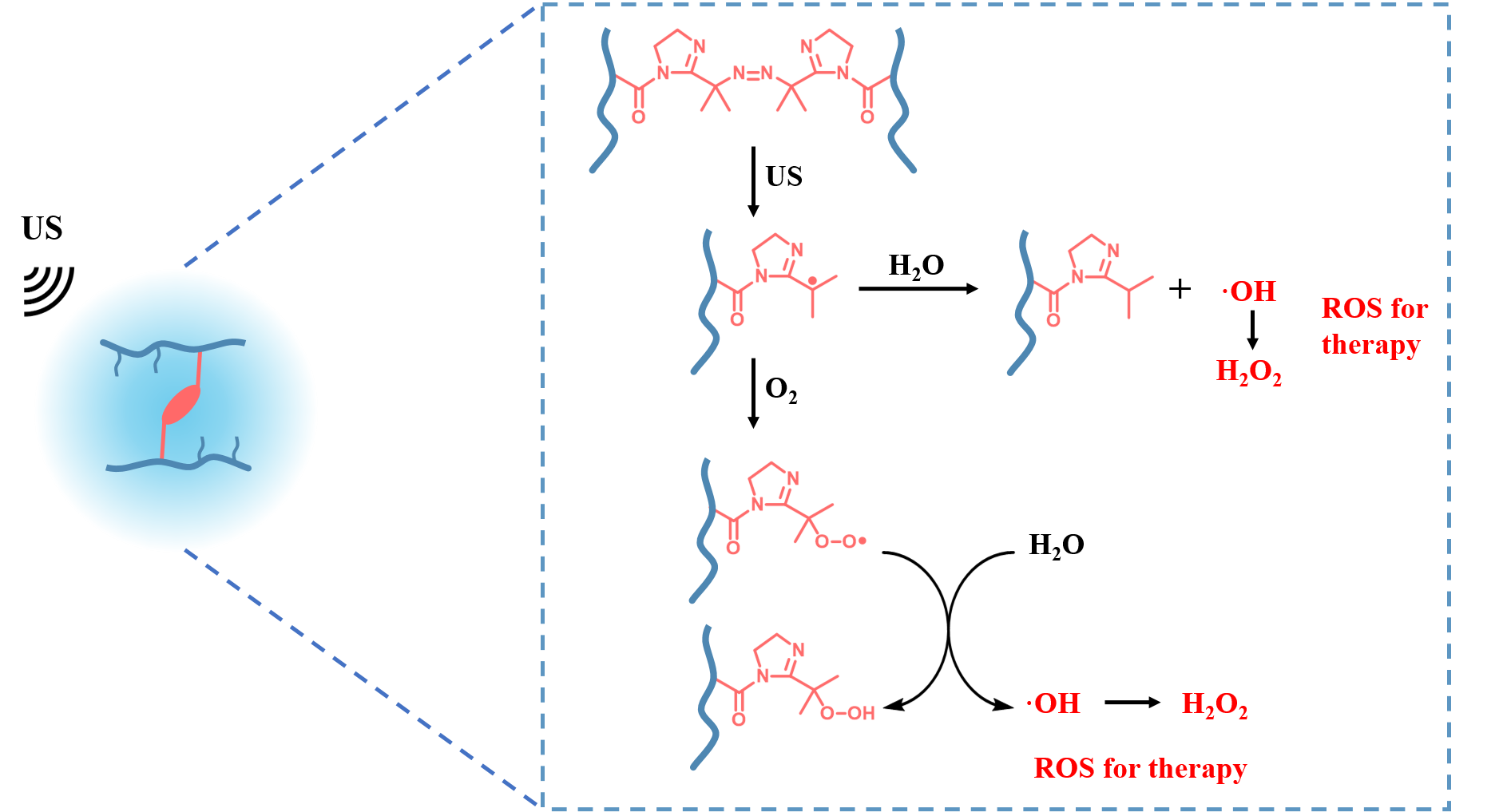


**Figure S47.** Mechanism of US-mediated mechanophore activation and subsequent ROS generation.

**Figure S48.** Mechanism of ABTS assay.^[1]^





**Figure S49.** Standard curve for calculating mechanophore activation efficiency.





**Figure S50.** Activation efficiency of mechanophores of PAP and NGs after treatment of LFUS for 5 min (n=3).





**Figure S51.** The UV absorbance at 734 nm of HS NGs under LFUS with different times.


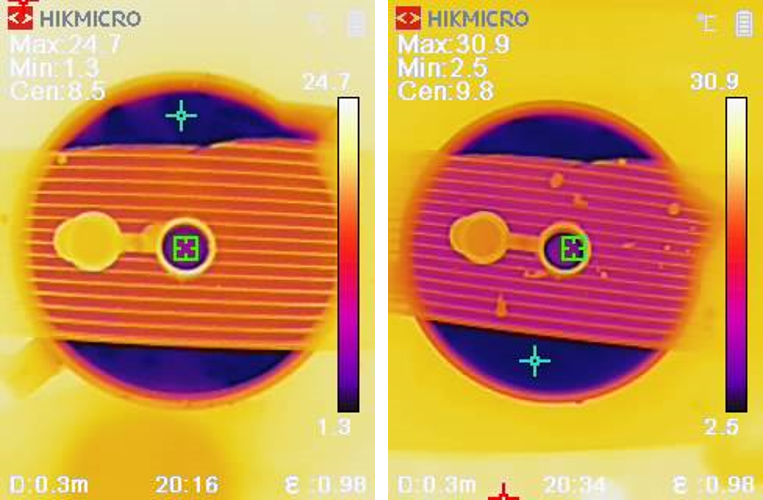


**Figure S52.** Infrared thermal images of HS NG solutions before (left) and after (right) treatment of LFUS for 5 min.


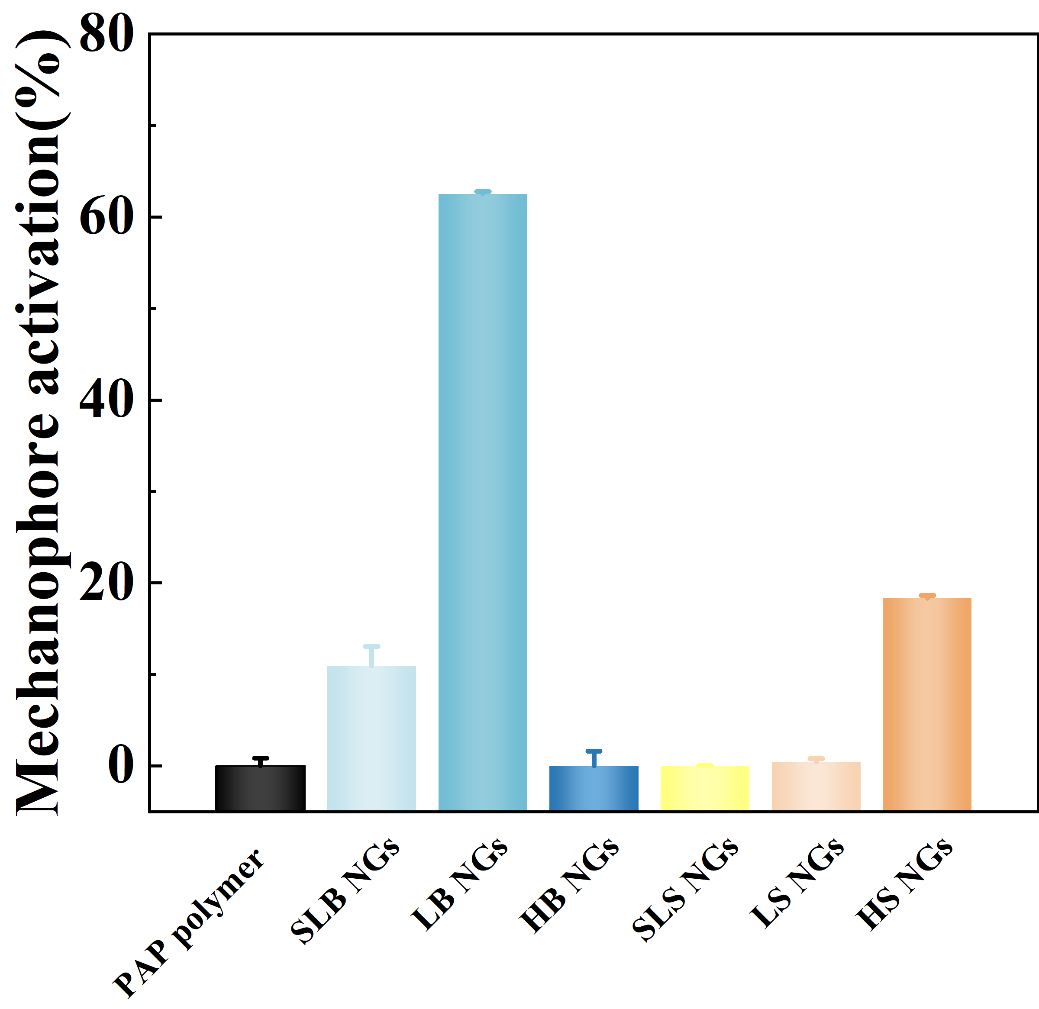


**Figure S53.** Activation efficiency of mechanophores of PAP and NGs after treatment of HFUS for 5 min (n=3).





**Figure S54.** The UV absorbance at 734 nm of LB NGs under HFUS with different times.


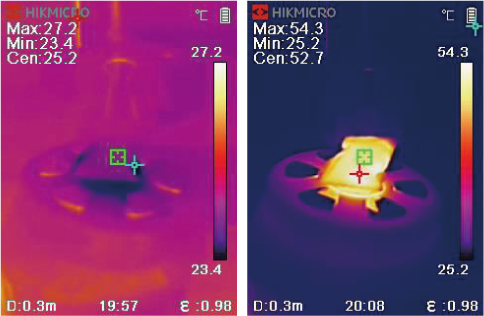


**Figure S55.** Infrared thermal images of LB NG solutions before (left) and after (right) treatment of HFUS for 5 min.





**Figure S56.** UV-vis spectra of PAP and NGs at 60 °C water bath for 5 min.





**Figure S57**. UV-vis spectra of LB-A polymer solutions containing ABTS after treatment of HFUS for 5 min.


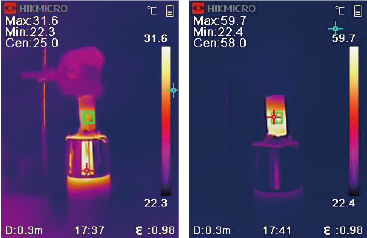


**Figure S58.** Infrared thermal images of NG solutions before (left) and after (right) treatment of HFUS with low intensity (2 W/cm^2^) for 3 min.


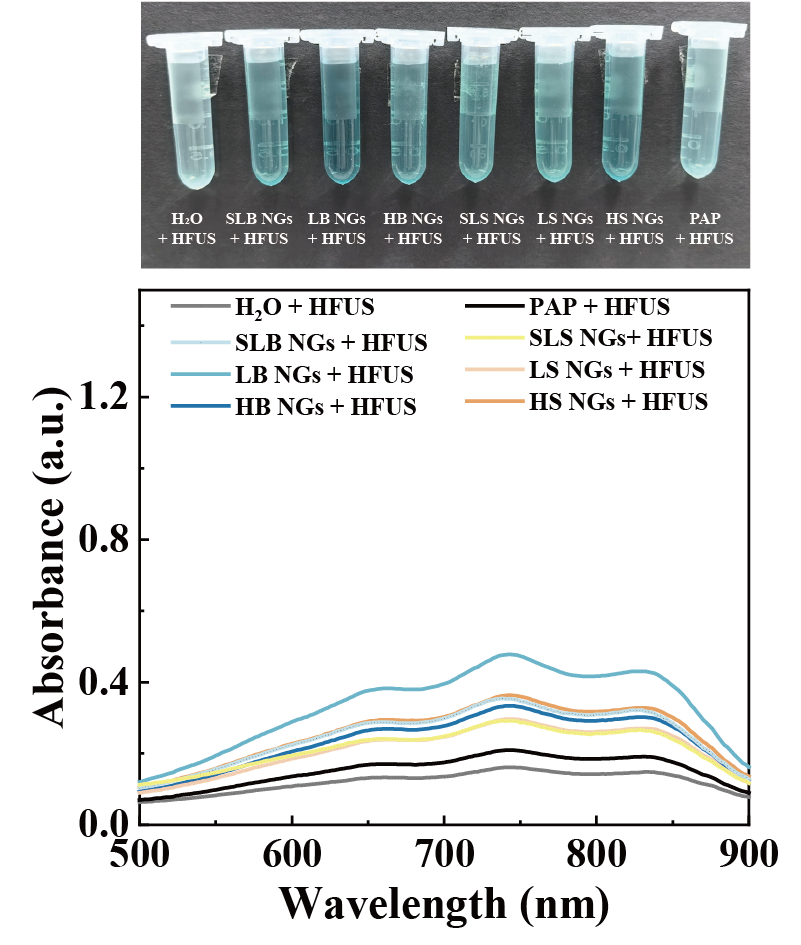


**Figure S59.** Photographs and UV-Vis spectra of PAP and NG solutions containing ABTS exposed to HFUS with low intensity (2 W/cm^2^) for 3 min.





**Figure S60.** UV-Vis spectra of LB NG solutions containing ABTS exposed to HFUS with low intensity (2 W/cm^2^) at different times (0-3 min).





**Figure S61.** Activation efficiency of mechanophores of PAP and NGs after treatment of HFUS with low intensity (2 W/cm^2^) for 3 min (n=3).

0
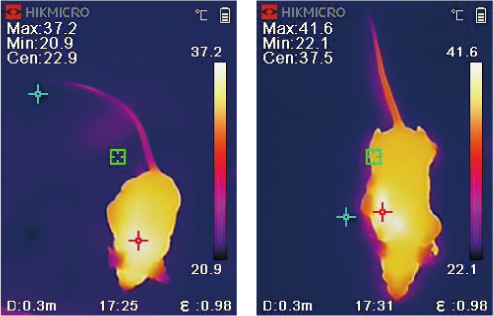


**Figure S62.** Infrared thermal images of nude mice before (left) and after (right) treatment of HFUS for 5 min.


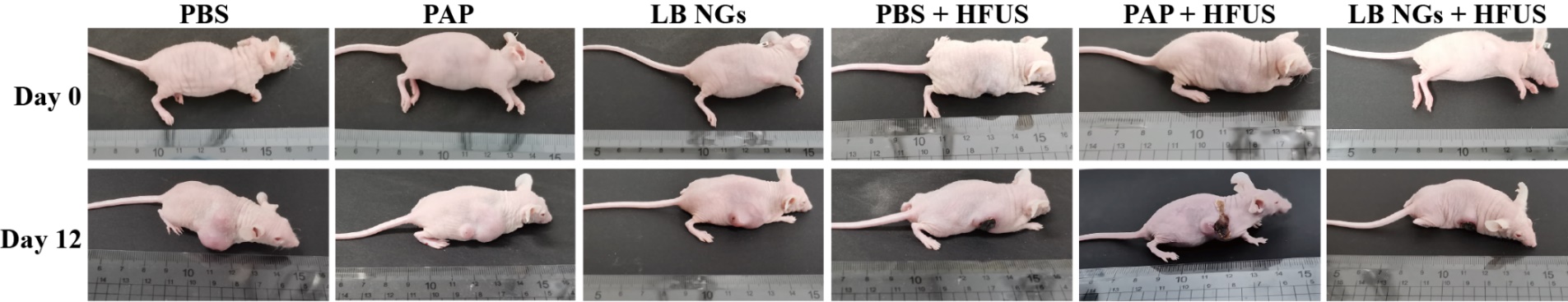


**Figure S63.** Photographs of the tumor-bearing mice before (day 0) and after different treatments (day 12).


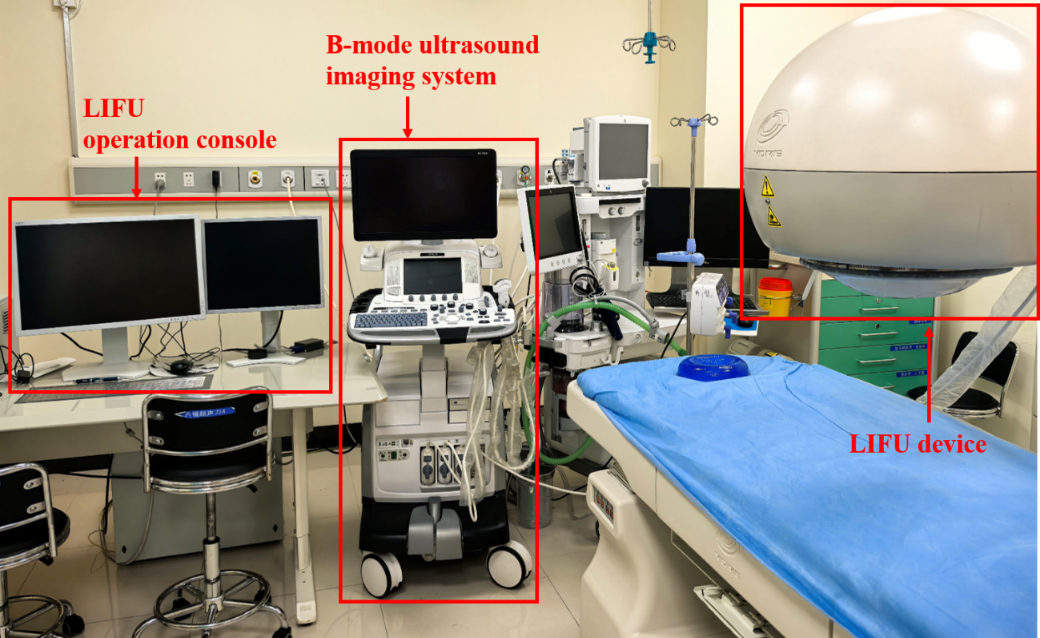


**Figure S64.** Overview of clinical LIFU system.


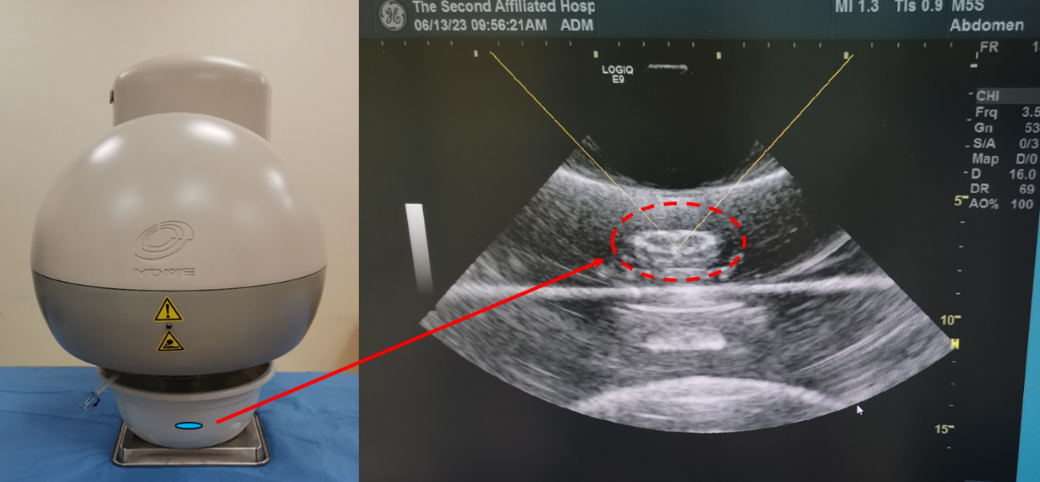


**Figure S65.** Photographs of clinical LIFU system for the mechanophore activation of NGs.


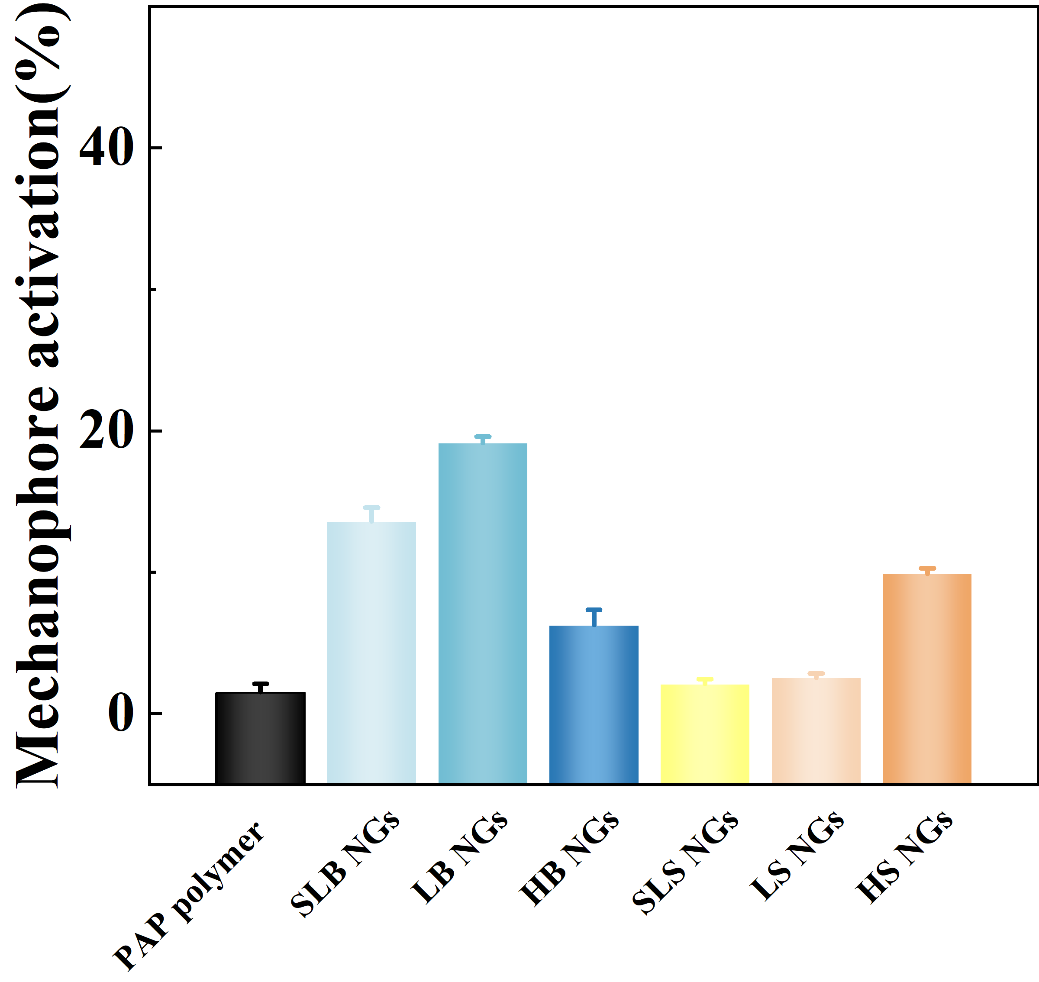


**Figure S66.** Activation efficiency of mechanophores of PAP and NGs after treatment of clinical LIFU for 5 min (n=3).


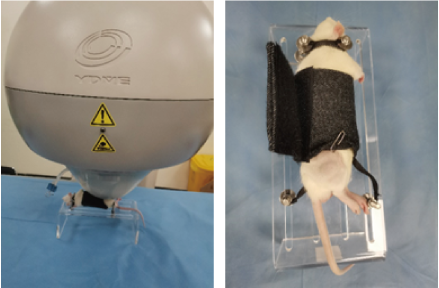


**Figure S67.** Clinical LIFU system for *in vivo* antitumor activity, and the rats were immobilized and placed under the clinical LIFU system with localization function (left). Rat immobilization device (right).


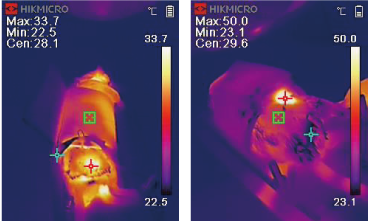


**Figure S68.** Infrared thermal images of rats before (left) and after (right) treatment of clinical LIFU.


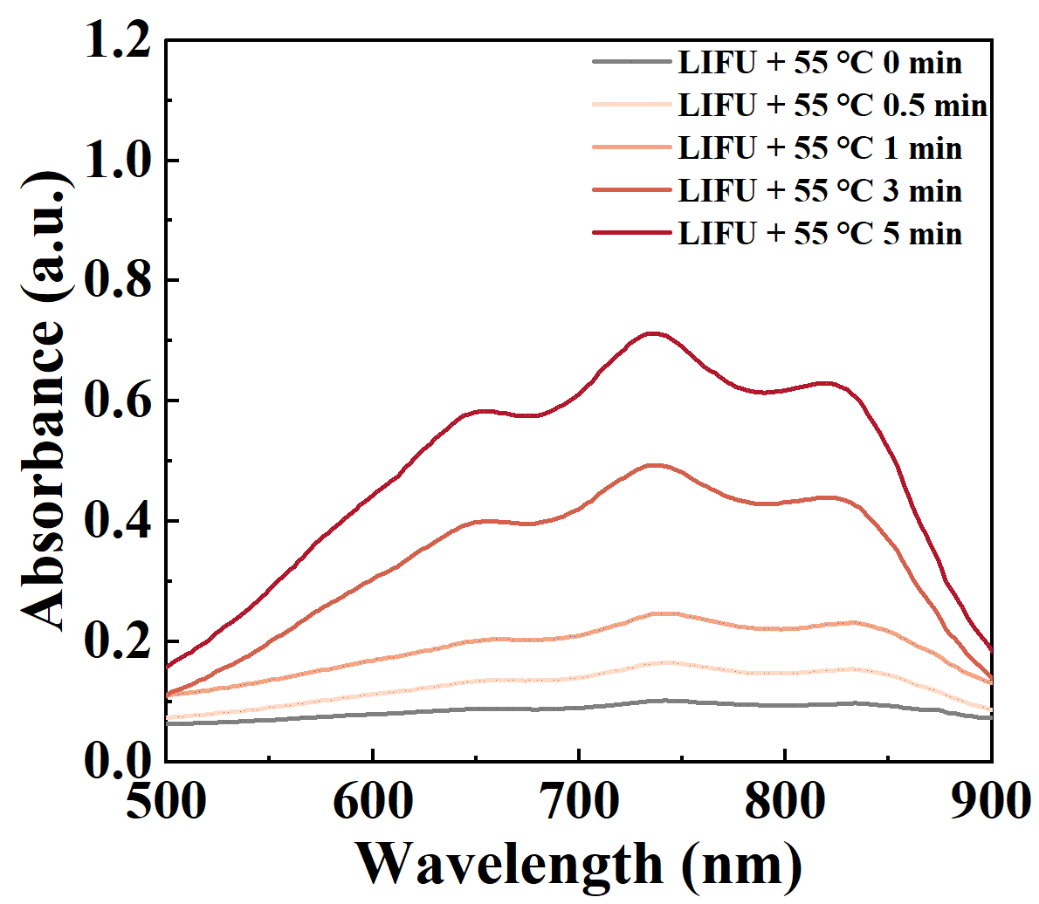


**Figure S69.** UV-Vis spectra of LB NGs containing ABTS and further exposure to LIFU in 55 °C water bath.


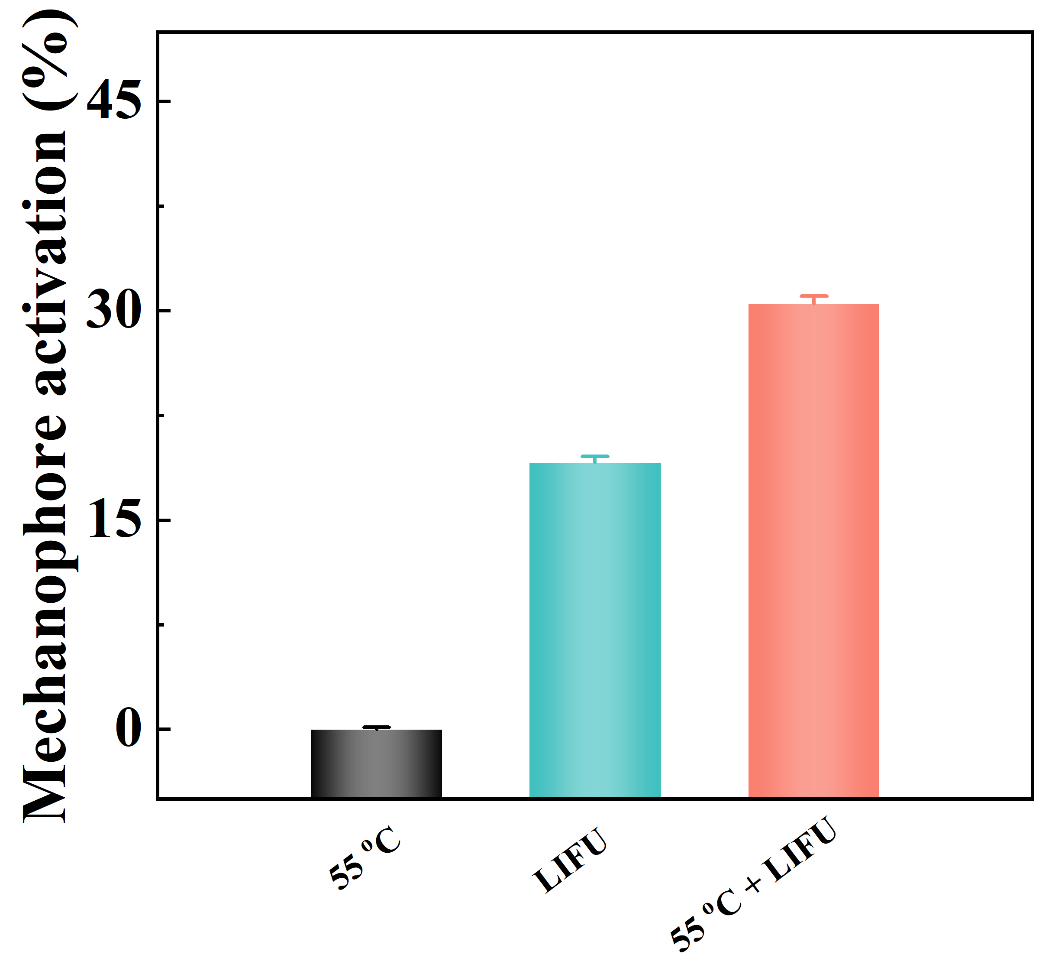


**Figure S70.** Activation efficiency of mechanophores of LB NGs after different treatments (n=3).

**References**

[1] a) E. P. Labrinea, C. A. Georgiou, *Analytica Chimica Acta* **2004**, *526*, 63-68; b) I. R. Ilyasov, V. L. Beloborodov, I. A. Selivanova, R. P. Terekhov, *International Journal of Molecular Sciences* **2020**, *21*, 1131.
